# Discovery of GuaB inhibitors with efficacy against *Acinetobacter baumannii* infection

**DOI:** 10.1128/mbio.00897-24

**Published:** 2024-08-29

**Authors:** Eric M. Kofoed, Ignacio Aliagas, Terry Crawford, Jialin Mao, Seth F. Harris, Min Xu, Shumei Wang, Ping Wu, Fang Ma, Kevin Clark, Jessica Sims, Yiming Xu, Yutian Peng, Elizabeth Skippington, Ying Yang, Janina Reeder, Savita Ubhayakar, Matt Baumgardner, Zhengyin Yan, Jacob Chen, Summer Park, Hua Zhang, Chun-Wan Yen, Maria Lorenzo, Nicholas Skelton, Xiaorong Liang, Liuxi Chen, Bridget Hoag, Chun Sing Li, Zhiguo Liu, John Wai, Xingrong Liu, Jun Liang, Man Wah Tan

**Affiliations:** 1 Department of Infectious Diseases, Genentech Inc., South San Francisco, California, USA; 2 Department of Discovery Chemistry, Genentech Inc., South San Francisco, California, USA; 3 Department of Medicinal Chemistry, Genentech Inc., South San Francisco, California, USA; 4 Department of Drug Metabolism and Pharmacokinetics, Genentech Inc., South San Francisco, California, USA; 5 Department of Structural Biology, Genentech Inc., South San Francisco, California, USA; 6 Department of Translational Immunology, Genentech Inc., South San Francisco, California, USA; 7 Department of Biochemistry and Cellular Pharmacology, Genentech Inc., South San Francisco, California, USA; 8 Department of Developmental Sciences Safety Assessment, Genentech Inc., South San Francisco, California, USA; 9 Department of Bioinformatics, Genentech Inc., South San Francisco, California, USA; 10 Department of Small Molecule Pharmaceutical Science, Genentech Inc., South San Francisco, California, USA; 11 Department of Protein Chemistry, Genentech Inc., South San Francisco, California, USA; 12 WuXi AppTec Co., Ltd., Shanghai, China; University of Pretoria, Pretoria, Gauteng, South Africa

**Keywords:** antibiotic target validation, structural biology, *Acinetobacter baumannii*, bacterial genetics, medicinal chemistry, pharmacology

## Abstract

Guanine nucleotides are required for growth and viability of cells due to their structural role in DNA and RNA, and their regulatory roles in translation, signal transduction, and cell division. The natural antibiotic mycophenolic acid (MPA) targets the rate-limiting step in *de novo* guanine nucleotide biosynthesis executed by inosine-5´-monophosphate dehydrogenase (IMPDH). MPA is used clinically as an immunosuppressant, but whether *in vivo* inhibition of bacterial IMPDH (GuaB) is a valid antibacterial strategy is controversial. Here, we describe the discovery of extremely potent small molecule GuaB inhibitors (GuaBi) specific to pathogenic bacteria with a low frequency of on-target spontaneous resistance and bactericidal efficacy *in vivo* against *Acinetobacter baumannii* mouse models of infection. The spectrum of GuaBi activity includes multidrug-resistant pathogens that are a critical priority of new antibiotic development. Co-crystal structures of *A. baumannii, Staphylococcus aureus*, and *Escherichia coli* GuaB proteins bound to inhibitors show comparable binding modes of GuaBi across species and identifies key binding site residues that are predictive of whole-cell activity across both Gram-positive and Gram-negative clades of Bacteria. The clear *in vivo* efficacy of these small molecule GuaB inhibitors in a model of *A. baumannii* infection validates GuaB as an essential antibiotic target.

**IMPORTANCE:**

The emergence of multidrug-resistant bacteria worldwide has renewed interest in discovering antibiotics with novel mechanism of action. For the first time ever, we demonstrate that pharmacological inhibition of *de novo* guanine biosynthesis is bactericidal in a mouse model of *Acinetobacter baumannii* infection. Structural analyses of novel inhibitors explain differences in biochemical and whole-cell activity across bacterial clades and underscore why this discovery may have broad translational impact on treatment of the most recalcitrant bacterial infections.

## INTRODUCTION

The discovery of antibiotics is arguably the most important medical advancement in human history, enabling the practice of modern medicine (1). However, the emergence of multidrug-resistant bacteria worldwide has raised the specter of pan-antibiotic resistance and has renewed interest in discovering therapies with novel mechanisms of action (2). Widespread resistance in *Enterococcus faecium*, *Staphylococcus aureus, Klebsiella pneumoniae*, *Acinetobacter baumannii*, *Pseudomonas aeruginosa*, and *Enterobacter* species has led to them being termed “ESKAPE” pathogens as they are the greatest concern to public health (3–5). New antibiotics that are effective against these diverse species of bacteria represent a large unmet medical need.

Mycophenolic acid (MPA) is a fungal natural product that was discovered to inhibit growth of the Gram-positive bacteria *Bacillus anthracis* (6) and *S. aureus* (7). Decades later, it was shown that MPA specifically inhibits the *de novo* guanine biosynthesis enzyme inosine-5´-monophosphate dehydrogenase (IMPDH) (GuaB in bacteria) (8). GuaB catalyzes the oxidation of the substrate inosine-5´-monophosphate (IMP) to xanthosine 5´-monophosphate (XMP) using NAD+ as a cofactor (9), and guanosine 5´-monophosphate (GMP) synthase converts XMP to guanosine 5'- monophosphate (GMP) to generate guanine nucleotides essential for cell growth and viability. Indeed, genetic studies have shown that *de novo* guanine biosynthesis is essential for growth and viability of bacteria both *in vitro* and *in vivo* (10–23).

Over the past 20 years, academic groups and pharmaceutical companies have investigated the possibility of targeting GuaB for use as an antibiotic, but several basic questions remain unanswered about whether such a strategy would work (9, 24–28). The primary controversy is over whether exogenous purines *in vivo* can be utilized by purine salvage mechanisms of bacteria during infection, therefore circumventing the need for *de novo* guanine biosynthesis (28).

Using sequence and structural analysis of the inhibitor binding pocket, we focused on an amino acid sequence signature that predicted intrinsic biochemical potency of GuaB inhibitors (GuaBi). This signature was first appreciated by Hedstrom et al. as providing selectivity over the human enzyme and could account for the difference in biochemical potency of GuaBi between *B. anthracis* GuaB and *Escherichia coli* GuaB (29–31). Phylogenetic analysis of these key residues across species, that we term “key selectivity” residues, predicted whole-cell activity of GuaBi against several ESKAPE pathogens (*E. faecium*, *S. aureus*, *A. baumannii*, *P. aeruginosa*), bacterial select agents (*Burkholderia),* and *Mycobacterium tuberculosis*. The vast majority of the bacteria kingdom contain these shared GuaB residues for which GuaBi exhibit whole-cell activity *in vitro*; however, divergence occurred within the Cyanobacteria phylum, Gammaproteobacteria class including *E. coli*, and the Peptostreptococcae family (32, 33). We hypothesize that species that contain divergent GuaB selectivity residues have reduced biochemical affinity for GuaBi by virtue of a strong desolvation penalty of more polar residues.

Cross-phylogeny conservation of the GuaB key selectivity residues encouraged us to test whether inhibitors designed to kill Gram-positive bacteria could also be effective against Gram-negative bacteria that share the matching motif. Attainment of whole-cell activity against Gram-negative bacteria is complicated by the intracellular location of the target separated by a dual membrane containing lipopolysaccharide and efflux machinery that limits the clinical use of Gram-positive antibiotics for Gram-negative infections. Structure-based, small molecule drug design, combined with the insight of the key selectivity hypothesis led to the development of novel and bacteria-selective GuaB inhibitors that exhibit *in vivo* efficacy against *A. baumannii*.

## RESULTS

### Genetic ablation of *guaB* is bactericidal in *A. baumannii in vitro* and *in vivo* thigh and lung models of infection

**Fig 1:**
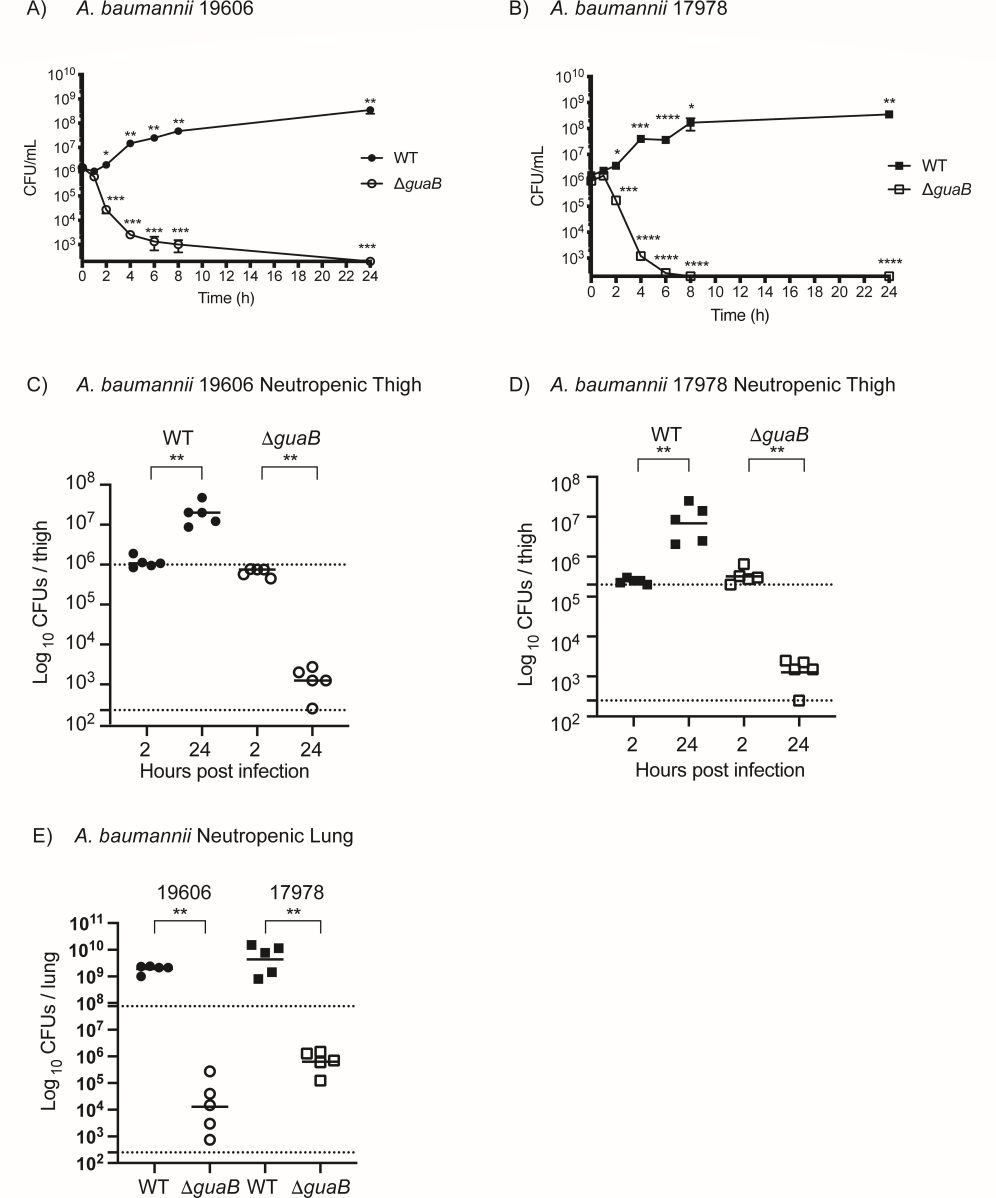
GuaB is essential for growth and viability of *Acinetobacter baumannii* both *in vitro* and *in vivo*. Wild-type and ΔguaB mutants of *A.b*. 19606 (**A**) and *A.b*. 17978 (**B**) were initially grown in guanine enriched media (M9 + 0.2% glucose + 1 mM guanine) and then shifted into media lacking guanine (M9 + 0.2% glucose) and colony forming units (CFUs/mL) were enumerated over time. Growth and survival of wild-type and ΔguaB mutants of *A.b*. 19606 (**C**) and *A.b*. 17978 (**D**) were evaluated in the murine neutropenic thigh model of infection, and in the murine neutropenic lung model of infection (**E**). CD1-Elite mice were used for neutropenic thigh models, and BALB/c mice were used for neutropenic lung models of infection. Statistical significance of experimental groups was determined using unpaired *t*-test comparing change from input of each strain (A and B; mean and SD; *n* = 3), or by unpaired non-parametric Mann-Whitney U-test (C–F; geometric mean; *n* = 5 mice per group) using GraphPad Prism.

To demonstrate the essentiality of *de novo* guanine biosynthesis in *A. baumannii*, *guaB* was deleted under permissible conditions in the presence of 100 µM guanine in two strains of *A. baumannii* (ATCC 19606 and 17978). When shifted into media lacking guanine, both *A. baumannii* Δ*guaB* mutants exhibited a 3–4 log decrease in number of viable bacteria within 6 h (Fig. 1A and B). Thus, *guaB* is essential in *A. baumannii* in the absence of exogenous guanine.

Due to the conditional essentiality of *guaB*, free guanine *in vivo* would allow *A. baumannii* Δ*guaB* mutants to remain viable *in vivo*. Therefore, we measured the concentrations of guanine, as well as the purine biosynthesis intermediates xanthine and guanosine, in mouse and human serum. Guanine [lower limit of quantification (LLOQ) = 1 µM] and guanosine (LLOQ = 0.034 µM) were both below the limit of detection in blood plasma from both human and mice, whereas xanthine was present at 0.36 ± 0.1 µM in human and 0.70 ± 0.55 µM in CD-1 mouse plasma (Table S1). The loss of viable *A. baumannii* Δ*guaB* in defined media was rescued by the addition of 100 µM exogenous guanine and xanthine, but failed to restore optimal growth of mutant bacteria (Fig. S1A and C). Continuously available high levels of exogenous guanine added to both liquid cultures and agar plates were required to completely rescue bacterial viability of ΔguaB mutants (Fig. S1B, D, E, and F). Thus, in normal, healthy mice and humans, there is insufficient circulating guanine to allow for growth of the *A. baumannii* Δ*guaB* mutants.

To directly assess the viability of the *A. baumannii* Δ*guaB* mutants *in vivo*, these mutants were tested in a mouse neutropenic thigh model of bacterial infection. For both strains of *A. baumannii*, the wild-type bacteria expanded 1–2 logs within 22 h of infection (Fig. 1C and D WT; *P* = 0.0079**). In contrast, deletion of *guaB* in both strains was bactericidal with a 2–3 log reduction in viable bacteria despite having equal number of bacteria in the thigh 2 h after injection (Fig. 1C and D Δ*guaB*; *P* = 0.0079**). These data show that GuaB is essential for viability and growth of *A. baumannii in vivo* in the context of neutropenic thigh model of infection. These *in vivo* essentiality findings were confirmed in a neutropenic mouse lung model of infection where deletion of *guaB* was bactericidal in both strains of *A. baumannii* (Fig. 1E Δ*guaB*; *P* = 0.0079**). Together, these data show that in two genetically distinct isolates and in two sites of infection, GuaB is essential for both growth and viability of *A. baumannii*. These data also suggest that free guanine, guanosine, and xanthine concentrations in lung and thigh tissue are insufficient to circumvent the requirement of *de novo* guanine biosynthesis during infection.

### Structure-based development of bacteria-selective small molecule inhibitors of GuaB

Many crystal structures of microbial GuaB/IMPDH have been solved to date, including co-crystal structures with small molecule inhibitors discovered from biochemical or phenotypic screens including *Cryptococcus neoformans* (34), *Cryptosporidium parvum* (29), *M. tuberculosis* (24, 31, 35–37), *P. aeruginosa* (38), *Campylobacter jejuni*, *Clostridium perfringens*, *Vibrio cholerae*, and *B. anthracis* (30). Medicinal chemistry efforts utilized these structural insights from these molecules to facilitate *de novo* design of new chemical scaffolds, leading to the identification of three highly potent compounds (G2, G1, and G8) with activity against a broad range of bacterial species and exquisite selectivity versus the human analogous enzyme IMPDH2.

**Fig 2:**
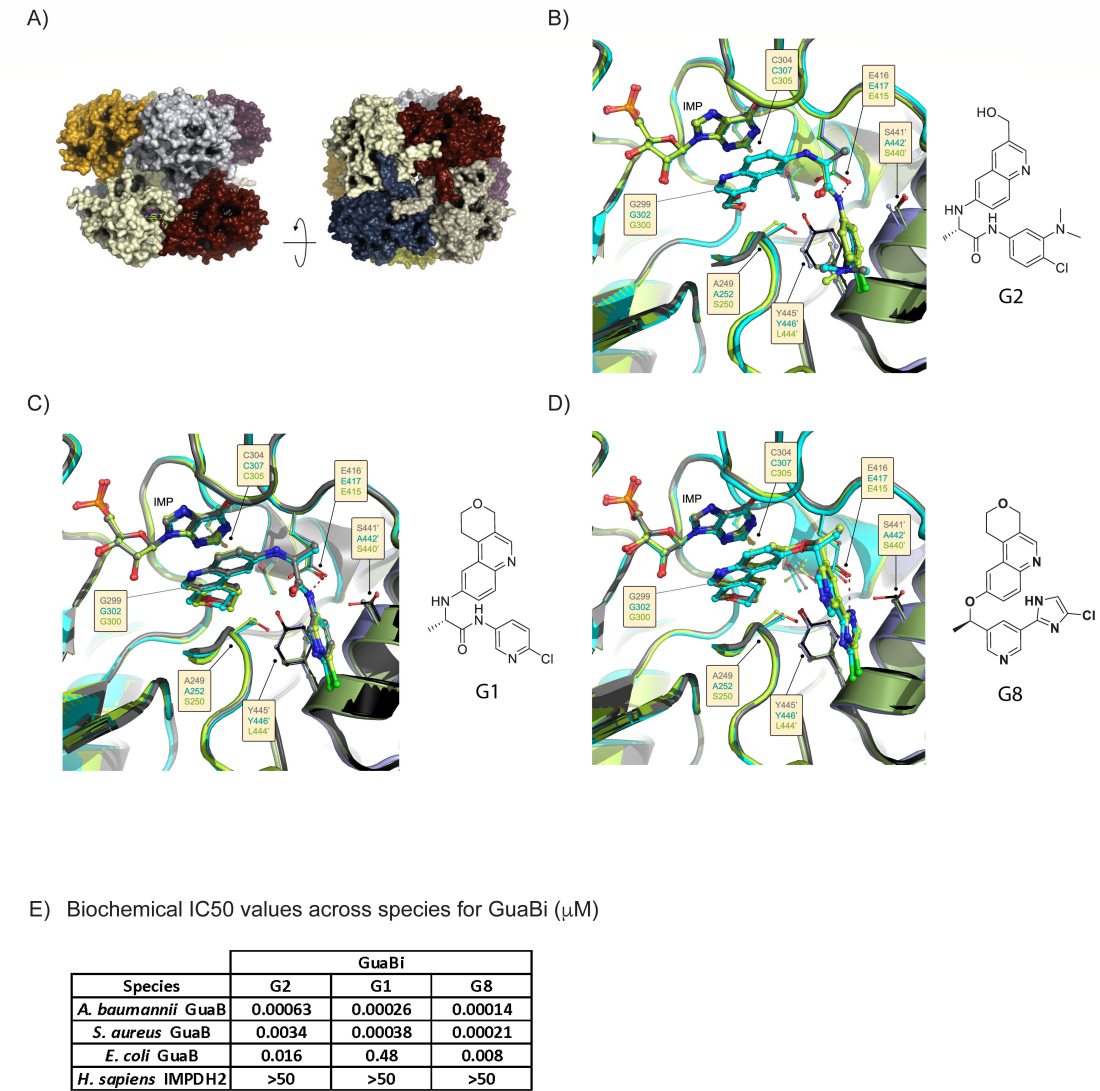
Higher order GuaB crystal structure and co-crystal structure overlays from *Acinetobacter baumannii*, *Staphylococcus aureus*, and *Escherichia coli* GuaB bound to novel inhibitors G2, G1, and G8. (**A**) GuaB protein showing tetramer and higher order octamer states. Co-crystal structure overlays of GuaB from *A. baumannii* (gray), *S. aureus* (teal), and *E. coli* (green) bound to G2 (**B**), G1 (**C**), and G8 (**D**). Biochemical IC50 values for G2, G1, and G8 for GuaB from *A. baumannii*, *S. aureus*, *E. coli*, and human IMPDH2.

Here, we present the first high resolution co-crystal structures of *A. baumannii*, *S. aureus,* and *E. coli* GuaB proteins (cystathionine beta-synthase (CBS) domains deleted), each determined with a matched set of three different inhibitors bound (Fig. 2; Table S2). The novel *S. aureus* and *E. coli* structures crystallize with one molecule in the asymmetric unit (asu), yet re-create the common GuaB tetramer arrangement via the fourfold symmetry operator of their I4 space group. The *A. baumannii* protein consistently crystallized in space group P21 with eight copies in the asu, forming an octamer composed of two inverted tetramers, relatively rotated to interdigitate the labile “finger” loops (residues 371–381) that mediate much of the tetramer:tetramer interface. Common to all three species’ structures, the amino- and carboxy-terminal ends of the protein are engaged in mutually reciprocal strand swaps with their neighboring protomer around the tetramer. Similarly, the NAD+ binding region, also where our compounds bind, occurs at the junction between neighboring molecules with side chains from both protomers contributing to ligand binding interactions. We observed a tight overlay of structures across the bacterial strains, highlighting common critical interactions required for potency. These inhibitors mimic the NADH/IMP cofactor interactions by forming strong π-π stacking interactions with IMP while also hydrogen bonding with the conserved glycine (Gly299^Ab^/302^Sa^/300^Ec^) which is one of the two hydrogen bond interactions observed with the NADH amide. The ligand linker segment induces a 90° bend, directing either the amide N-H of G2 and G1 or the imidazole of G8 to form an important hydrogen bond to the conserved glutamic acid (Glu416^Ab^/417^Sa^/415^Ec^) while directing the aryl tail group into a hydrophobic shelf region. This Glu residue underpins species selectivity, because the human homologs (IMPDH1/2) contain a Gln residue at this position that effectively prevents binding. However, these interactions do not fully explain the discrepancy in biochemical activity observed between the three species (Fig. 2E).

Structural analysis of the key selectivity region of the proteins provides a rationale for the observed intrinsic biochemical potency of the GuaBi across species. Differences in the polarity of a particular key selectivity residue specifically (Ser250 in *E. coli* versus Ala252 in *S. aureus* and Ala249 in *A. baumannii*) was found to be critical for inhibitor interaction with GuaB which corroborates the observations of previous reports (29, 30). As shown in Fig. S2, the key residues (SER-440 and SER-250) in the binding site of GuaB *E. coli* contribute to a stable and extensive water network (more green and well-connected spheres) predicted by WaterMap analysis with the pseudo-apo crystal structures. To disrupt such water network, the compound needs to pay a larger desolvation penalty leading to poor potency, whereas the same compound binding to GuaB in *S. aureus* and *A. baumannii* does not need to pay for such penalty with a less favorable water network (more red and less connected spheres). Based on our analysis of the water networks present in these structures, we hypothesize that differences in the free energy of solvation of the binding site produce the observed difference in binding activity (Fig. 2E).

Enzymatic mechanism of inhibition studies showed GuaBi binds to *A. baumannii* GuaB uncompetitively with respect to IMP, and via a mixed non-competitive binding type with respect to NAD+(Fig. S3). Uncompetitive inhibitors are desirable because they retain inhibition independent of substrate concentration, and can bind both E-IMP and E-XMP complexes.

### Cross-phylogeny conservation of key selectivity residues and membrane architecture determine biochemical and whole-cell activity of GuaBi

We wanted to expand on the key selectivity residue structural motif to determine if we could predict *a priori* if a particular bacterial species would be sensitive to GuaBi. Potent minimal inhibitory concentrations (MICs) were obtained for all the Gram-positive bacteria examined, and all possess an Ala at the first differentiating position and Tyr at the second coupled position (Table 1). However, Gram-negative bacteria whole-cell activity is complicated by the fact that they have a dual membrane containing lipopolysaccharide and efflux machinery that reduce the concentration of inhibitor at the site of the intracellular target (39). Despite these complications, our hypothesis was confirmed as the clades of Gram-negative bacteria that retain the conserved key selectivity residues in the GuaB protein were still sensitive to the GuaBi in the low micromolar range, whereas *Enterobacteriaceae* that contain the divergent residues were resistant (Table 1).

**TABLE 1:** Antibiograms of GuaBi minimal inhibitory concentration (MIC) across bacterial species showing the correlation of the key selectivity residues to whole-cell activity and the impact of the Gram-negative outer membrane^*a*^

Species	Gram stain	Average MIC (μM)			Coupled residue pair			
		G1	G2	G8	Coupled residue 1	Coupled residue 2 on alpha-helix	Alpha-helix minus 4 position	Coupled residue pair
*Staphylococcus aureus*	+	0.1	1.6	0.01	Ala252	Tyr446	Ala442	A/Y
*Staphylococcus epidermidis*	+	0.03	0.4	0.004	Ala252	Tyr446	Ala442	A/Y
*Mycobacterium avium*	Variable	0.8	12.5	0.04	Ala285	Tyr489	Ala485	A/Y
*Mycobacterium tuberculosis* H37Rv	Variable	0.3	2.5	0.2	Ala285	Tyr487	Ala483	A/Y
*Mycobacterium marinum*	Variable	0.01	0.1	0.03	Ala288	Tyr490	Ala486	A/Y
*Mycobacterium smegmatis*	Variable	0.01	1.3	0.02	Ala320	Tyr522	Ala518	A/Y
*Enterococcus faecium*	+	1	5.2	0.03	Ala255	Tyr450	Ser446	A/Y
*Enterococcus faecalis*	+	0.2	0.3	0.01	Ala259	Tyr454	Ser450	A/Y
*Bacillus subtilis*	+	1.2	13.8	0.05	Ala253	Tyr445	Ser441	A/Y
*Pseudomonas aeruginosa*	−	100	>100	50	Ala249	Tyr446	Ala442	A/Y
*Stenotrophomonas maltophilia*	−	14	38	5	Ala250	Tyr443	Ala439	A/Y
*Burkholderia cepacia*	−	67	>100	3.1	Ala249	Tyr443	Ala439	A/Y
*Burkholderia thailandensis* E264	−	25	100	1.6	Ala250	Tyr444	Ala440	A/Y
*Acinetobacter baumannii*	−	0.5	3.1	1.3	Ala249	Tyr445	Ser441	A/Y
*Escherichia coli*	−	>100	>100	>100	Ser250	Leu444	Ser440	S/L
*Citrobacter werkmanii*	−	>100	>100	>100	Ser250	Leu444	Ser440	S/L
*Serratia marcescens*	−	>100	>100	>100	Ser250	Leu444	Ser440	S/L
*Enterobacter cloacae*	−	>100	>100	>100	Ser250	Leu445	Ser441	S/L
*Enterobacter aerogenes*	−	>100	>100	>100	Ser250	Leu444	Ser440	S/L
*Klebsiella pneumoniae*	−	>100	>100	>100	Ser250	Leu444	Ser440	S/L
*Proteus mirabilis*	−	>100	>100	>100	Ser250	Leu444	Ser440	S/L
Human IMPDH1	n.a.	n.a.	n.a.	n.a.	Ser276	Asp470	His466	Other
Human IMPDH2	n.a.	n.a.	n.a.	n.a.	Ser276	Asp470	His466	Other

^*a*^ The GuaB coupled residue pair A/Y is associated with whole-cell activity, whereas the S/L pair is associated with intrinsic biochemical resistance to GuaBi and lack of whole-cell activity. MICs were performed using the Clinical & Laboratory Standards Institute microdilution method with modification of the broth and culture duration (see Materials and Methods “MICs”). Key selectivity residues were identified by multiple sequence alignments using the CLUSTAL_MUSCLE software. Bacterial MIC assay and Gram stain are not applicable (n.a.) to the purified human enzyme.

**Fig 3:**
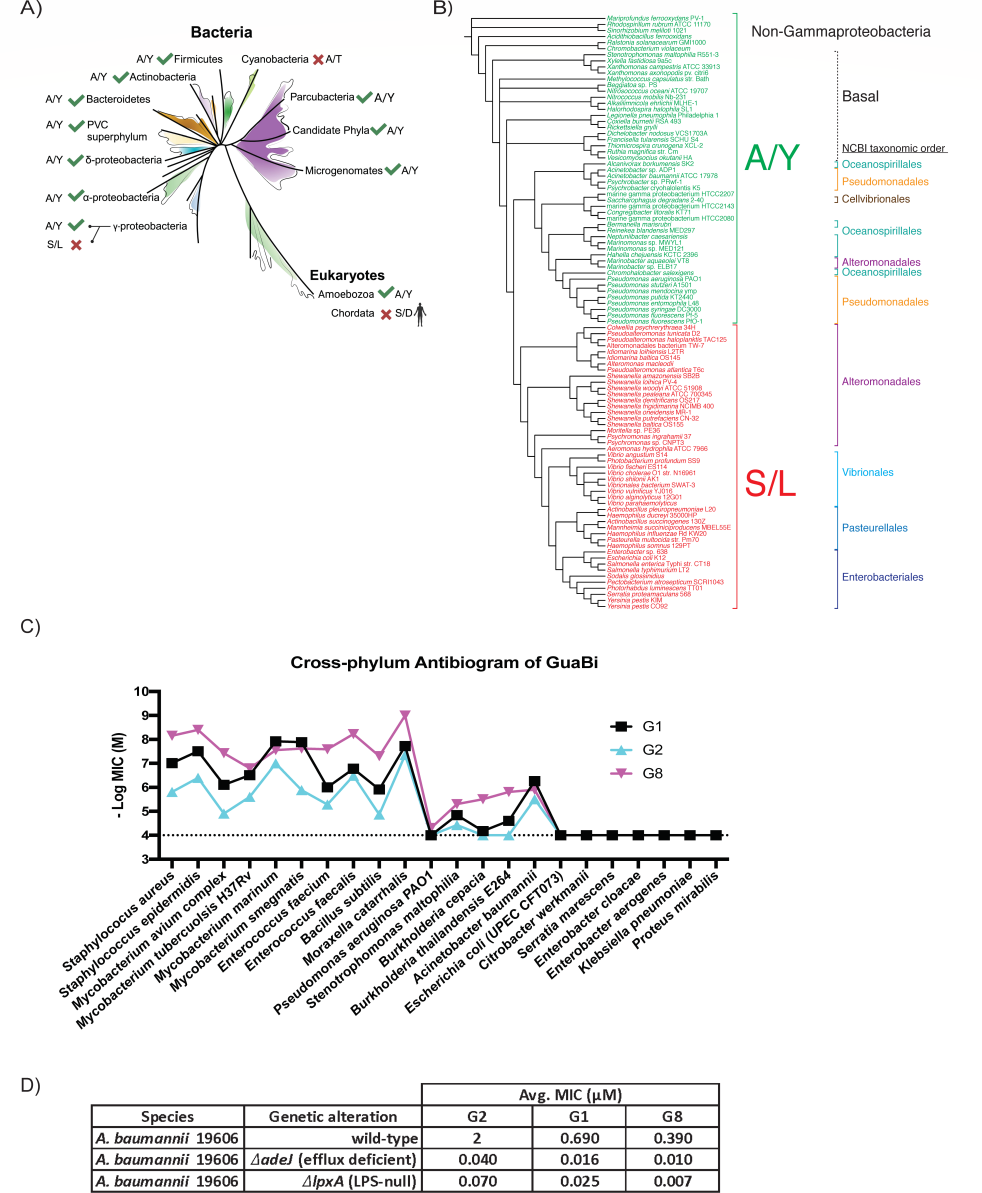
Cross-phylogeny conservation of key selectivity residues and membrane architecture determine biochemical and whole-cell activity of GuaBi. (**A**) Phylogeny of microbial life showing broad conservation of GuaB A/Y residue pairing (green check) and isolated clades of divergence (red X) based on the reproduced phylogeny of Hug et al. (32) and alignments of representative species (Table S3) (Created with Biorender.com). (**B**) The distribution of A/Y and S/L key selectivity residues in 92 gammaproteobacterial and six outgroup genomes. The phylogeny shown is a previously published maximum likelihood-based estimate of relationships among these bacteria (33). (**C**) Antibiogram of GuaBi G1, G2, and G8 MIC across bacteria species. (**D**) MIC of GuaBi G2, G1, and G8 on *A. baumannii* 19606 (wild-type), *A. baumannii* Δ*lpxA* (LPS-null), and *A. baumannii* Δ*adeJ* (efflux-defective). Dotted line in (**C**) reflects the top concentration (100 µM) of the MIC assays (>100 µM for all wild-type S/L pairing species).

Interestingly, the configuration of the A/Y key selectivity residues are widely conserved across the bacterial kingdom with few exceptions (Fig. 3A) (32). Divergence at these key sites occurred within the Cyanobacteria phylum, the Gammaproteobacteria class including *E. coli*, and the Peptostreptococcae family (Fig. 3A and B; Table S3) (32, 33). Despite the challenges of outer membrane permeability and efflux of small molecules by Gram-negative bacteria, GuaBi retained whole-cell activity against a wide variety of important human pathogens including *A. baumannii* (Fig. 3C). As expected, high level of exogenous guanine rescued growth in culture of *A.b*. 19606 treated with G1, G2, and G8 that confirms on-target specificity of the inhibitors (Table S4). However, the introduction of gene deletions that compromise efflux (Δ*adeJ*) and lipopolysaccharide biosynthesis (Δ*lpxA*) renders *A. baumannii* hyper-sensitive to GuaBi, confirming the important contribution of both efflux and lipopolysaccharide (LPS) to whole-cell activity (Fig. 3D). Altogether, in Gram-negative bacteria, the cumulative penalties of outer-membrane penetration, efflux, and the desolvation of key selectivity residues fully explains the resistance of particular species like *E. coli* to GuaBi.

### Metabolism and pharmacokinetics of G2 and G1 in mouse

Motivated by the *in vitro* potency of GuaBi against *A. baumannii*, we explored the potential of these antibacterial molecules *in vivo*. Pharmacokinetic (PK) properties of the GuaBi were tested to enable *in vivo* efficacy testing. G2 had high clearance in mouse hepatocytes. However, metabolism of G2 was inhibited by 38% by introduction of 1-aminobenzotriazole (ABT), a non-isoform-specific and time-dependent cytochrome P450 (CYP) inhibitor, and 4-methylpyrazole (4-MP), inhibitor of alcohol dehydrogenase. In a 24-h i.v. infusion, the exposure of 750 mg/kg G2 was found to be 21,300 (µM * h, AUC_0-24_) when co-administered with 150 mg/kg ABT and 100 mg/kg 4-MP in mice, which is 100-fold higher than without co-administration with ABT and 4-MP (Fig. S4A). The unbound fraction of G2 in human, rat, and mouse plasma were 0.045%, 0.074%, and 0.12%.

Similarly, although G1 had high clearance in mouse hepatocytes, its metabolism was effectively inhibited by 39% by 1 mM ABT. In the 24-h i.v. infusion, the exposure of 250 mg/kg G1 was found to be 692 (µM * h, AUC_0-24_) when co-administered with 150 mg/kg ABT in mice, which is fourfold higher than without the co-administration with ABT (Fig. S4B). The unbound fraction of G1 in human, rat, and mouse plasma were 0.055%, 0.02%, and 0.051%.

Overall, the *in vitro* safety profiles of both G2 and G1 indicated good selectivity, low promiscuity, and low cytotoxicity, enabling efficacy studies, whereas G8 had a high promiscuity risk, off-target selectivity, and potential hERG and cytotoxicity liabilities, precluding testing this compound *in vivo* (Table S5).

### Pharmacologic inhibition of GuaB is bactericidal in neutropenic thigh models of infection against *A. baumannii*

**Fig 4:**
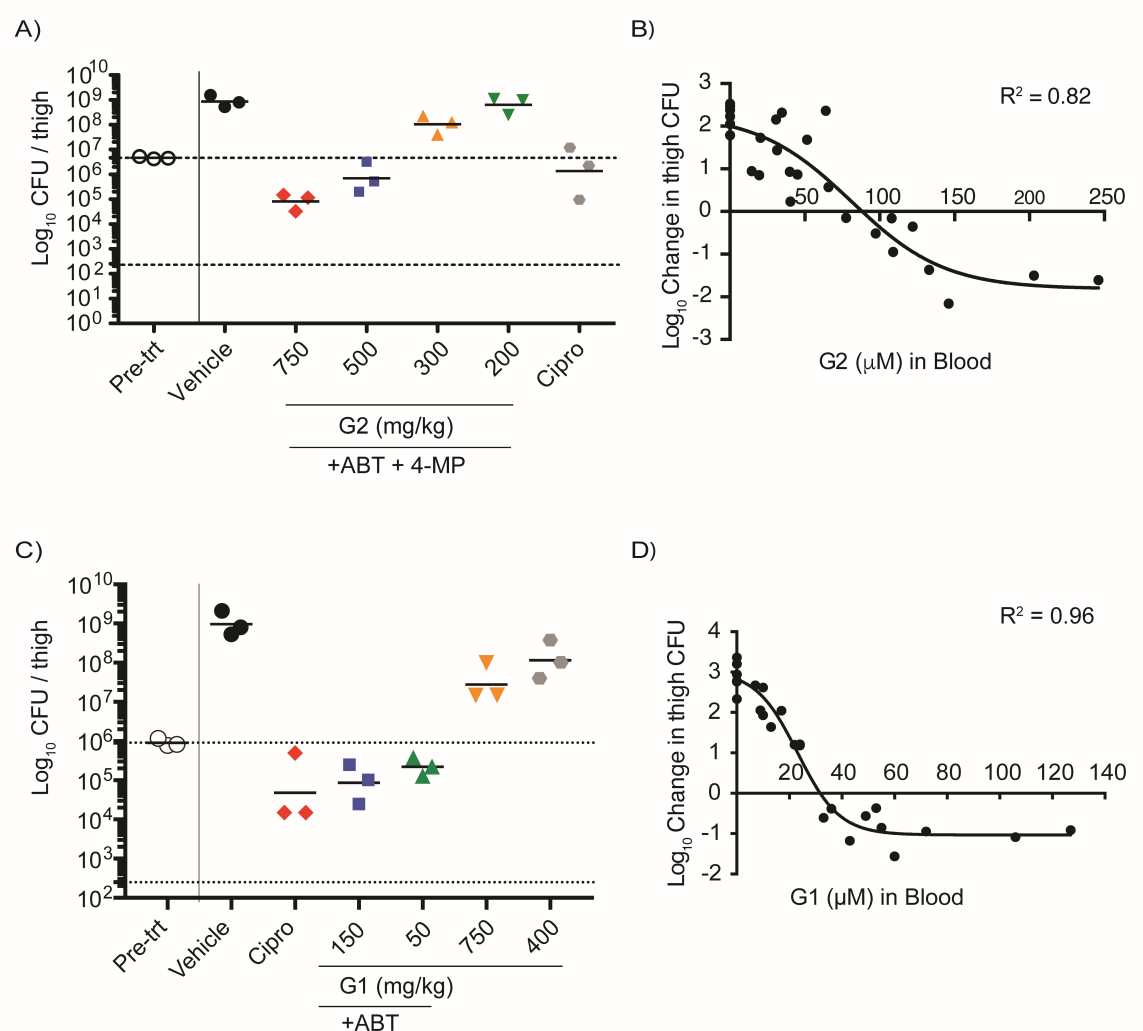
*In vivo* efficacy of GuaBi G2 and G1 against *A. baumannii* in the murine neutropenic thigh model of infection. (**A**) Neutropenic CD-1 mice were infected with *A. baumannii* 19606 in the thigh muscle followed by intravenous delivery of vehicle or escalating doses of G2 in combination with 1-aminobenzotriazole (150 mg/kg) and 4-MP (100 mg/kg) delivered orally, or ciprofloxacin for 24 h, and bacterial load was enumerated by plating for colony forming units (CFUs/thigh muscle). (**B**) Pharmacodynamic response of bacterial load as a function of G2 concentration in blood at 5 h plotted to the right. (**C**) Neutropenic CD-1 mice were infected with *A. baumannii* 19606 in the thigh muscle, followed by intravenous delivery of vehicle or escalating doses of G1 alone or in combination with 1-aminobenzotriazole (500 mg/kg BID or QD) delivered orally, or ciprofloxacin for 24 h, and bacterial load was enumerated, and (**D**) pharmacodynamics were plotted as described in (**B**) above. Pharmacodynamic plots are combined data from two independent experiments including all dosing groups, and each dot represents data from a single mouse.

To determine whether pharmacologic inhibition of GuaB *in vivo* recapitulates the genetic phenotype of Δ*guaB* mutants *in vivo,* we used a neutropenic thigh model of *A. baumannii* infection therapeutically treated with i.v. administered GuaBi G2 and G1. GuaBi exhibited dose-dependent bactericidal efficacy against *A. baumannii in vivo* (Fig. 4). Both G2 (Fig. 4A) and G1 (Fig. 4C) reduced the bacterial load in thigh muscle compared to pre-treatment in both high dose groups. Change in bacterial load per thigh muscle plotted against the blood concentration of G2 (Fig. 4B) and G1 (Fig. 4D) showed efficacy within a few multiples of the serum MIC and pharmacokinetic & pharmacodynamic (PK-PD) target attainment. Additionally, we show that the free fraction of drug (unbound to protein) over the serum-free MIC accurately predicts *in vivo* efficacy (Fig. S5). G2 was bactericidal against *A. baumannii* at 5× F_u_/MIC (Fig. S5B), and G1 was bactericidal above 2× F_u_/MIC (Fig. S5D). The free fraction of drug determines the concentration of inhibitor able to distribute into tissues from the blood and engage the target within the bacteria, and is an important factor that impacts the pharmacodynamics of antibiotics (40). Thus, G2 and G1 have demonstrated *in vivo* antibacterial activity that can be predicted from *in vitro* activity, making them attractive lead compounds for optimization.

### GuaBi have low frequency of on-target spontaneous resistance

Drug resistance is a major problem that hinders the efficacy of standard of care antibiotics against pathogenic bacterial infections (4), and the frequency of resistance for any new antibiotic candidate is an important characteristic that will ultimately determine its clinical success (41). The importance of resistance frequency to clinical efficacy is highlighted by recent failures during phase IIb clinical trials that demanded termination of the trial (42). To determine the frequency of resistance to the novel GuaBi, we performed fluctuation analysis (43) by plating 20 parallel cultures of *A. baumannii* onto 20 agar plates containing 4× MIC or 8× MIC of GuaBi. After 3 days of growth at 37°C, we counted the number of resistant mutants per plate and calculated the frequency of resistance using the p0 method (44). GuaBi-resistant colonies were re-streaked onto GuaBi containing plates to verify stability of the phenotype, and the GuaB target gene and 500 bp flanking regions were fully sequenced to identify the mutation that underlies the GuaBi-resistant phenotype (Table S6). All GuaBi-resistant mutants sequenced were found to have on-target single nucleotide missense mutations in the *guaB* open reading frame. Mutations in *A.b*. GuaB Y445S/N/C and *A.b*.GuaB P25A/S are predicted to reduce the affinity of GuaBi-binding to the enzyme based on their location in the GuaB inhibitor binding site, but further studies are required to confirm this hypothesis. These on-target mutations were obtained at low frequency for both G2 (3.6 × 10^−10^ to 5.1 × 10^−10^) and G1 (6.4 × 10^−11^ to 2.9 × 10^−10^), but did confer high-level resistance of with ≥32-fold shifts in MIC (Table S6). Mutational cross-resistance profile was determined for *A.b*. 19606 Y445N/C/S and P25A/S mutants and ranged in effect from 2.5× to >200× MIC (Table S7). GuaBi binding to conserved residues within the catalytic site is believed to underlie the low frequency of resistance.

### GuaB inhibitors are active against carbapenem-resistant *A. baumannii* clinical isolates

To determine the activity of GuaB inhibitors against clinically relevant circulating populations of *A. baumannii*, we obtained panels of carbapenem-resistant clinical isolates from the Centers for Disease Control and Prevention (CDC) and International Health Management Associates (IHMA) and performed broth MICs using the Clinical & Laboratory Standards Institute (CLSI) protocol. The MIC_50_ and MIC_90_ for G2 was 6.25 µM and 16 µM, respectively, and for G1 was 3.1 µM and 8 µM, respectively (Fig. S6). A two- to threefold difference in MIC_50_ and MIC_90_ values and the normal distribution of MIC values against the isolates suggest that there is no pre-existing resistance mechanism against GuaBi in circulating clinical isolates.

## DISCUSSION

Here, we demonstrate the utility of targeting *de novo* guanine biosynthesis as an antibacterial therapy for *A. baumannii* infection *in vivo* by generation of potent GuaB inhibitors guided by rational structure-based drug design. Bactericidal activity of the GuaBi *in vivo* against *A. baumannii* recapitulates the genetic data and reveals a path forward for therapeutic intervention in the *de novo* guanine biosynthetic pathway. Data presented here show that free exogenous purine concentrations *in vivo* are insufficient to rescue viability of *A. baumannii* in both *guaB* genetic ablation and GuaB pharmacologic inhibition experiments. Importantly, GuaB is essential for growth and viability of *A. baumannii* both in neutropenic thigh and lung models of infection, the lung representing the natural site of infection (Fig. 1). The risk of subverting GuaB essentiality by purine salvage is allayed by the observation that purine rescue requires orders of magnitude more guanine than is available during *in vivo* infection (Fig. S1; Table S1). In the neutropenic thigh model of infection, which translates well in the clinic for antibiotic development, nucleobase permeases/transporters are not sufficient to compensate for the loss to GuaB activity in *A. baumannii* (Fig. 1 and 4).

The most important goal for preclinical development of GuaBi is to improve the metabolic profile so that maintenance of blood pharmacokinetics above the Fu/MIC is achievable without the use of PK boosters like ABT and 4-MP. G1 was designed specifically to remove the hydroxy-alcohol that is a substrate of extra-hepatic alcohol-dehydrogenase metabolism, and retained excellent biochemical and *in vivo* potency. However, more work on this scaffold is required to reduce the cytochrome P450 metabolic liability to enable single agent dosing in future preclinical animal models.

Together, the data presented here show the value of combining bacterial genetics with structure-based drug design to develop a potent new class of antibiotic active against *A. baumannii*. GuaBi are bactericidal and bacteria-selective with a low frequency of spontaneous resistance. Furthermore, the conservation of GuaBi binding mode to the enzyme across clades of the bacteria kingdom in combination with knowledge of the key selectivity residues gives us the power to predict *a priori* which enzymes will be intrinsically sensitive to inhibition by GuaBi. The prediction is realized as whole-cell activity against a large number of extremely important bacterial pathogens that have an outsized impact on human morbidity and mortality in clinics across the globe.

## MATERIALS AND METHODS

### Bacterial isolates used in this study

*Acinetobacter baumannii* 19606 and 17978 were obtained from ATCC. *A. baumannii* clinical isolates used in MIC_50_ and MIC_90_ determination were obtained from the CDC (0045, 0063, 0037, 0052, 0056, 0036, 0035, 0033, 0101, 0101, 0102, 0083, 0078, 0070) and IHMA (872842, 925928, 941383, 979816, 951461, 941161, 929995, 952682, 945295, 945295, 937775, 927970, 965318, 945483, 869388, 945062, 1326091, 1104756, 1251405, 1244158, 1094444, 1297652). Strains used in MIC testing of GuaBi were *S. aureus* (USA300 NRS384), *Staphylococcus epidermidis* RP62A (ATCC35984), *Mycobacterium avium* (ATCC25291), *Mycobacterium tuberculosis* H37Rv (ATCC27294), *Mycobacterium smegmatis* (mc2-155 (ATCC700084), *Enterococcus faecium* (ATCC700221), *Enterococcus faecalis* (ATCC47077), *Bacillus subtilis* (Ward’s Bioscience 85W1640), *Pseudomonas aeruginosa* PAO1 (ATCC-BAA-47), *Stenotrophomonas maltophilia* (ATCC700267), *Burkholderia cepacia* (ATCC25416), *Burkholderia thailandensis* E264 (ATCC700388), *Escherichia coli* UPEC CFT073 (ATCC700928), *Citrobacter werkmanii* (ATCC51114), *Serratia marcescens* (ATCC29021), *Enterobacter cloacae* (ATCC222), *Enterobacter aerogenes* (ATCC13048), *Klebsiella pneumoniae* (ATCC 43816), and *Proteus mirabilis* (ATCC29906).

### Genetic manipulations of *Acinetobacter baumannii*

Genetic deletions were generated in *A. baumannii strains* ATCC 19606 and ATCC 17978 using the Rec_Ab_ recombinase one-step chromosomal gene inactivation system (45). Briefly, the open reading frames of *guaB*, *lpxA*, and *adeJ* were replaced with a kanamycin-resistance cassette by recombineering. The kanamycin-resistance cassette was amplified off the pACYC177 vector and spliced to 500 bp flanking regions of each target gene and electroporated into bacteria carrying the Rec_Ab_ plasmid and selected for recombinants. Mutants were verified by colony PCR using outside primers and full sequencing of the genomic region.

### MICs

MICs were determined according to the CLSI microdilution method starting with 5 × 10^5^ colony forming units (CFU)/mL inoculum grown for 20 h incubation at 37°C with modification of the broth to M9 base + 0.5% casamino acids + 0.2% glucose and read visually for *A. baumannii*, *E. coli*, *P. aeruginosa*, *S. maltophilia*, *B. cepacia*, *B. thailandensis*, *C. werkmanii*, *S. marcescens*, *E. cloacae*, *E. aerogenes*, *K. pneumoniae*, and *P. mirabilis*. MICs for *S. aureus*, *S. epidermidis*, *E. faecium*, *E. faecalis*, and *B. subtilis* were performed using the method above except using RPMI1640 media. MICs for *Mycobacterium marinum* and *M. smegmatis* were performed as above using Middlebrook 7H9 + OADC supplement (Sigma) grown for 48 h or 14 days for *M. avium* at 37°C. MICs for *M. tuberculosis* H37Rv were performed by TICRO biosciences grown in 7H9 + OADC broth for 8 days and read using the resazurin redox assay. MICs are reported as the average of at least three independent experiments.

### Time kill assays

The wild-type *A. baumannii* strains and the corresponding Δ*guaB* strains were revived from −80°C frozen stocks and grown on M9 agar plates supplemented with 500 µM guanine for 18 h. An inoculum size of 1.0 × 10^6^ CFU/mL was washed with M9 liquid medium to remove residual guanine and grown at 37°C. Aliquots of 0.2 mL of the culture were taken at time intervals of 0, 2, 4, 6, 8, and 24 h. The CFU of the bacteria was determined by serial dilution and growing on M9 agar plates supplemented with 500 µM guanine for 18 h. The procedure was performed in triplicate (three independent experiments).

### Animal usage declaration

All animal procedures were conducted under protocols approved by the Genentech Institutional Animal Care and Use Committee in an Association for Assessment and Accreditation of Laboratory Animal Care (AAALAC)-accredited facility in accordance with the Guide for the Care and Use of Laboratory Animals and applicable laws and regulations.

### Neutropenic thigh infection model

CD-1 mice (Charles River Laboratories) were rendered neutropenic by two intraperitoneal injections of cyclophosphamide (Baxter Health Care Corporation) at 150 mg/kg on day −5 and 100 mg/kg on day −2. On day 0, 50 µL log-phase grown bacteria, *Acinetobacter baumannii* (ATCC 19606 and 17978) 2 × 10^5^ CFU/mouse and GuaB deletion mutants 2 × 10^6^ CFU/mouse, was injected into the mouse thigh muscle. At 24 h post infection, CFU was determined in the thigh muscle through serial dilutions.

### Neutropenic lung infection model

BALB/c mice (Charles River Laboratories) were rendered neutropenic by two intraperitoneal injections of cyclophosphamide (Baxter Health Care Corporation) at 150 mg/kg on day −4 and 100 mg/kg on day −1. On day 0, mice were infected intranasally with 40 µL log-phase grown *Acinetobacter baumannii* (ATCC 19606, 17978, or GuaB deletion mutants) at 1 × 10^8^ CFU/mouse. At 24 h post infection, CFU was determined in the lung through serial dilutions.

### Neutropenic thigh i.v. delivery of GuaBi and co-dosing with PK boosters

Jugular vein-catheterized CD-1 mice (Charles River Laboratories) were rendered neutropenic by two intraperitoneal injections of cyclophosphamide (Baxter Health Care Corporation) at 150 mg/kg on day −5 and 100 mg/kg on day −2. On day 0, 50 µL log-phase grown bacteria, *Acinetobacter baumannii* (ATCC 19606) 1 × 10^6^ CFU/mouse, was injected into the mouse thigh muscle. A P.O. dose of cytochrome P450 nonspecific inhibitor 1-aminobenzotriazole at 150 mg/kg was given right after infection. At 2 h post infection, different doses of GuaB inhibitor G2 and 100 mg/kg 4-MP co-formulated in 5% dimethyl sulfoxide (DMSO) with 3% Cremophor EL and 2.5% of ethanol in saline were intravenously infused at 80 µL per hour for 22 h. Ciprofloxacin (Claris Lifesciences Inc.) was dosed at 80 mg/kg subcutaneously once at 2 h post infection. At 24 h post infection, CFU was determined in the thigh muscle through serial dilutions.

### Fluctuation assays

Fluctuation studies were performed using 20 independent cultures of *A. baumannii* 19606 picked from 20 independent colonies grown to late log phase in 20 mL M9 media supplemented with 100 µM guanine, bacteria pelleted by centrifugation at 4,500 rpm for 10 min, washed twice with phosphate-buffered saline (PBS), and plated onto M9 + casamino acids + 0.2% glucose agar plates containing 4× or 8× MIC of GuaBi. Agar plates with GuaBi were prepared by mixing agar with compounds at 50°C prior to pouring plates. Selection was performed by growing bacteria at 37°C, 5% CO_2_, for 3 days, followed by counting the number of resistant colonies on each plate and applying the p0 method to derive the spontaneous mutation rate (46). Resistant mutants were re-streaked on GuaBi containing agar plates, picked for growth in 3 mL M9 broth, and frozen at −80°C in 20% glycerol:80% bacterial M9 culture.

### Identification of GuaBi-resistance mutations

Single colonies of GuaBi-resistant bacteria from the re-streaked fluctuation assay were resuspended in 100 µL colony PCR buffer (50% NEB Cutsmart buffer) in a PCR tube, boiled at 99°C for 5 min, and 2 µL of this genomic template was used to amplify the genomic *guaB* open reading frame including 500 bp flanking regions. Amplicons from each mutant were sequenced entirely using Sanger DNA sequencing using *A.b. guaB* sequencing primers.

### GuaB sequence analysis

The *E. coli* K-12 MG1655 GuaB sequence was compared by TBLASTN (47) to 97 gammaproteobacterial and six non-gammaproteobacterial genomes downloaded from GenBank (http://www.ncbi.nlm.nih.gov/). The genomes were selected to sample a previously published phylogeny of *Gammaproteobacteria* (33). In total, 98 complete GuaB sequences were identified based on inspection of BLAST alignments satisfying e-value <0.001. Protein sequences were extracted from GenBank and aligned using MAFFT (48) with the local pairwise sequence alignment with the affine gap cost (L-INS-I) option, and the distribution of A/Y and S/L key selectivity residues was determined.

### Mechanism of GuaB enzyme inhibition

Mechanism of inhibition determination absorbance assays were carried out using *A. baumannii* 19606 GuaB protein and varied substrate concentrations in 384 well black clear bottom plates (Corning 781091). The indicated final reaction concentrations of IMP (7.5, 15, 30, 60, 120, 240, 480, and 960 µM) at fixed concentration of 1,000 µM NAD or NAD (18.75, 37.5, 75, 150, 300, and 600 µM) at fixed concentration of 500 µM IMP were combined in assay buffer (Tris pH 8, 100 mM KCl, 3 mM EDTA, 0.002% Tween-20, 1 mM DTT) in a total volume of 50 µL per well. Substrates were added to assay ready plates containing 250 nL of compound which had been previously diluted as an 11-point serial dilution in DMSO with a top concentration of 2 µM. Additional DMSO was added as a backfill (final DMSO 1% vol/vol). Compound addition and backfill were preformed using a Labcyte Echo 550. The reaction was initiated by the addition of 10 µL of *A. baumannii* GuaB enzyme (5 nM–30 nM). Reaction plate was spun using an Agilent Velocity 11 Microplate Centrifuge VSpin (1,800 rpm, 30 s), then the absorbance at 340 nm was measured for 1 h (intervals of 1 min 30 s) using a SpectraMax M5 (ambient). Initial velocity was taken as the initial linear slope for each time course. The data were fitted globally using GraFit Data Analysis Software (Erithacus Software) according to an uncompetitive/mixed type inhibition model obeying the two-substrate sequential kinetics.

### hERG 2pt patch clamp assay

All chemicals used in solution preparation were purchased from Sigma-Aldrich (St. Louis, MO) unless otherwise noted. Test article and positive control concentrations were prepared fresh daily by diluting stock solutions into a HEPES-buffered physiological saline solution (composition in mM): NaCl, 137; KCl, 4.0; CaCl_2_, 1.8; MgCl_2_, 1; HEPES, 10; glucose, 10; pH adjusted to 7.4 with NaOH. HEK293 cells were stably transfected with hERG cDNA and maintained in the culture medium with the appropriate selection pressure and antibiotics. All experiments were performed at room temperature. Test articles were evaluated at the concentrations of 1 µM and 10 µM, and cisapride was used as the positive control.

In preparation for a recording session, intracellular solution was loaded into the intracellular compartments of the QPlate and cell suspension was pipetted into the extracellular compartments. After establishment of a whole-cell configuration, membrane currents were recorded using up to 48 parallel patch clamp amplifiers in the QPatch HT system. The current records were sampled at 2,000 Hz and low-pass Bessel filtered at 400 Hz. Onset and block of hERG current were measured using a stimulus voltage pattern consisting of a 500 ms prepulse to –40 mV (leakage subtraction), a 2-s activating pulse to +40 mV, followed by a 2-s test pulse to −40 mV. The pulse pattern was repeated continuously at 10-s intervals, from a holding potential of −80 mV. Peak tail current (upper panel) was measured during the −40 mV test pulse. Leakage current was calculated from the current amplitude evoked by the prepulse and subtracted from the total membrane current record. Data acquisition and analysis was performed using the QPatch Assay Software (Sophion Bioscience A/S, Denmark). Solubility of all compounds was measured using TurboSol in conjunction with the ion channel testing.

### Secondary pharmacology panel

All assays were performed at Cerep Eurofins. The affinity of the test article for 41 different cellular targets was evaluated. For instance, the affinity of the test article for dopamine D1 receptor was assayed as follows: cell membrane homogenates were incubated for 60 min at 22°C with 0.3 nM [3H]SCH 23390 in the absence or presence of the test compound in a buffer containing 50 mM Tris-HCl (pH 7.4), 5 mM KCl, 5 mM MgCl_2_, 1.5 mM CaCl_2_, and 5 mM EDTA. Nonspecific binding was determined in the presence of 1 µM SCH 23390. Following incubation, the samples were filtered rapidly under vacuum through glass fiber filters (GF/B, Packard) presoaked with 0.3% poly(ethyleneimine)(PEI) solution and rinsed several times with ice-cold 50 mM Tris-HCl using a 96-sample cell harvester (Unifilter, Packard). The filters were dried then counted for radioactivity in a scintillation counter (Topcount, Packard) using a scintillation cocktail (Microscint 0, Packard). The results were expressed as a percent inhibition of the control radioligand specific binding. The standard reference compound is SCH 23390, which is tested in each experiment at several concentrations to obtain a competition curve from which its IC50 is calculated.

### 2-Day primary human hepatocyte cytotoxicity assay

Primary human hepatocytes were plated on 384-well, collagen-coated plates in incubation media with no serum or with 10% fetal bovine serum (FBS). Eighteen hours later, cells were treated with the test article at seven concentrations starting at 100 µM in twofold dilutions, chlorpromazine (positive control) at seven concentrations starting at 100 µM in twofold dilutions or aflatoxin (positive control) at seven concentrations starting at 25 µM in twofold dilutions. Cell viability was then assayed using the Promega Cell TiterGlo ATP Assay.

### Pharmacokinetics and metabolism of G2 and G1

#### *In vitro* metabolism studies in mouse hepatocytes

The incubation of G2 and G1 with cryopreserved mouse hepatocytes was conducted according to the standard in-house protocol. Cryopreserved hepatocytes were thawed using pre-warmed InVitroGRO HT thawing medium in 50 mL centrifuge tubes. The tubes were centrifuged for 3 min at 50 × *g*, and the supernatants were discarded. Cells were resuspended with 50 mL of pre-warmed Dulbecco’s modified eagle medium (DMEM) by gently inverting the tube several times. The tubes were centrifuged for 3 min at 50 × *g*, and the supernatants were discarded. Cells were brought up in 5.0 mL of pre-warmed DMEM incubation medium. The total cell counts and the number of viable cells were determined by the trypan blue exclusion method. Incubations were carried out in 20 mL scintillation vials containing 1.0 mL of hepatocyte suspension (approximately 1.3 × 10^6^ cells/mL). The mouse hepatocytes were pre-incubated with or without 1 mM ABT or 4-MP for 15 min. Vials were spiked with G2 or G1 as a DMSO stock solution to a final concentration of 5 µM G2 or G1 and a final DMSO concentration of 0.1%. The scintillation vials were placed on an orbital shaker rotating at 16 rpm in a humidified incubator at 37°C, 5% CO_2_. The reaction was quenched with 4 mL of acetonitrile at 0 or 3 h. The hepatocyte samples were protein precipitated with 4 volumes of acetonitrile and centrifuged at 2,000 × *g* for 5 min. The resulting supernatant was reduced to a dry residue and reconstituted with 350 µL water:acetonitrile (2/1; vol/vol). All samples were analyzed by LC-MS/MS system.

Liquid chromatography (LC) equipment consisted of a CBM 20A Communications Module, NexeraX2 LC 30AD pumps, NexeraX2 SIL 30AC autosampler, CTO Prominence Column Oven from Shimadzu (Columbia, MD), and a 1290 Infinity DAD ultraviolet (UV) detector with a 60 mm flow cell from Agilent Technologies (Waldbronn, Germany). The LC column was an EVO C_18_ (1.9 µm, 2.1 × 100 mm) from Phenomenex. Mobile phase A was water with 0.1% formic acid and mobile phase B was acetonitrile with 0.1% formic acid. The flow rate was 400 µL/min and the injection volume was 10 µL. Column temperature was 40°C. Mass spectrometry was performed using a 5600 QTRAP mass spectrometer equipped with a TurboIonSpray from Applied Biosystems (Redwood City, CA). Various tandem mass spectrometry (MS/MS) functionalities were used for analysis, which included data-dependent time-of-flight (TOF) scans in a positive ion electrospray mode. The extracted ion chromatography traces were obtained following analysis of mouse hepatocyte incubations and were used to quantify the metabolites.

#### *In vitro* plasma protein binding study

Plasma protein binding experiments were performed in triplicate (*n* = 3) using a Single-Use Rapid Equilibrium Dialysis (RED) plate from Thermo Fisher Scientific Inc. (Rockford, IL) by following the standard protocol from the supplier. Initially, test compounds were spiked to plasma (pH 7.4) to achieve a final concentration of 5 µM, and aliquots of 300 µL drug-plasma mixtures were then transferred to the donor wells of the RED plate which was pre-loaded with 500 µL phosphate buffer saline (133 mM) on the receiver wells. The RED plate was sealed with a gas permeable membrane and placed in a shaking incubator (450 rpm, VWR Symphony) for 6 h at 37°C with 5% CO_2_. At the end of incubation, aliquots of 30 µL samples were taken out from the receiver wells and donor wells, and matrix was equalized by adding an equal volume of plasma or buffer, respectively. Subsequently, resulting samples were quenched with 3 volumes of ice-cold acetonitrile containing propranolol (0.1 µM) as the internal standard to precipitate plasma protein. After shaking for 15 min at 500 rpm on a Thermo Scientific Compact Digital MicroPlate Shaker, all samples were then subjected to centrifugation at 3,700 rpm for 15 min (Beckman Coulter Allegra X 12R) to remove precipitated protein, and supernatants were then collected for LC-MS/MS analyses after dilution with an equal volume of water.

LC-MS/MS analysis was performed using a 6500+ QTRAP mass spectrometer coupled with a TurboIonSpray ESI ion source (AB SCIEX, Redwood City, CA) and Shimadzu Nexera X2 UPLC (Kyoto, Japan). Chromatographic separation of all analytes was achieved using a Kinetex C18 column (30 × 2.1 mm, 100 Å, 2.6 µm particle size) (Torrance, CA) along with mobile phase A consisting of 0.1% formic acid in high-performance liquid chromatography (HPLC) grade water and mobile phase B consisting of 0.1% formic acid in acetonitrile. A generic LC gradient was used for all analytes where the flow rate was set to 1 mL/min, the run time at 2 min, and the LC gradient as follows: 3% B for the first 0.1 min, ramped up to 95% B from 0.10 to 0.35 min, remained constant at 95% B for 0.50 min, and then decreased to 3% B within 0.65 min. Fraction of unbound drug (fu) was calculated using MS peak area ratios of test compound to IS in buffer and plasma wells, respectively.

#### *In vivo* pharmacokinetic study in mouse

An i.v. formulation of 5% DMSO with 3% Cremophor EL and 2.5% of ethanol in saline was used for all studies. Two female CD-1 mice were given a 750 mg/kg of 24 h i.v. infusion dose of G2. Another group of two mice received 750 mg/kg of G2 and 100 mg/kg 4-MP (co-dosed) 24-h i.v. infusion dose with a 150 mg/kg ABT PO dose of pretreatment at 2 h prior the i.v. infusion. Two female CD-1 mice were given a 250 mg/kg of 24-h i.v. infusion dose of G1. Another group of two mice received 250 mg/kg of 24-h i.v. infusion dose of G1 with a 150 mg/kg ABT PO dose of pretreatment at 2 h prior the i.v. infusion. Ten microliters of blood was collected accurately at 0.5, 1, 2, 4, 6, 8, and 24 h after infusion start. Blood samples were then immediately placed into labeled polypropylene micro-centrifuge tubes containing 40 µL of 1.7 mg/mL potassium (K2) EDTA in water, mixed, and then frozen and stored at −70°C until bio-analysis.

### Bio-analysis

G2, G1, ABT, and 4-MP blood concentrations in mouse PK studies were determined by LC-MS/MS. All samples were prepared by protein precipitation. Chromatographic separations were achieved using an ACQUITY CM UPLC system (Waters, Milford, MA) including an ACQUITY BSM solvent delivery system, an ACQUITY SM autosampler, and an ACQUITY CM column oven. For G1, G2, and ABT, an ACQUITY UPLC HSS T3 column (2.1 × 50 mm, 1.8 µm) was used. For 4-MP, an ACQUITY UPLC BEH Amide column (2.1 × 50 mm, 1.7 µm) was used. Gradient elution that contained 0.1% formic acid in water and 0.1% formic acid in acetonitrile was used for G1, ABT, and 4-MP. Gradient elution that contained 0.1% formic acid with 2 mM ammonium formate in water/acetonitrile (vol/vol, 95:5) was used for G2. The LC flow rate was 0.6 mL/min for G1, G2, and ABT, and 0.5 mL/min for 4-MP. The sample injection volume was 1 µL for ABT, G1, and G2, and 2 µL for 4-MP. A QTrap 5500/6500 tandem mass spectrometer (SCIEX, Foster City, CA) with TurboIonSpray interface was operated in positive ionization mode with multiple reaction monitoring (MRM) for LC-MS/MS analysis. The mass spectrometer was operated at unit mass resolution for both Q1 and Q3 quadrupoles. The precursor to product ion transitions of analytes are presented as Q1/Q3 ratios for each compound: 4-MP (83.0/56.1), G1 (383.1/197.1), G2 (399.1/171.0), and ABT (135.0/80.1). The standard curves cover the ranges of 0.005 µg/mL–11.7 µg/mL, 0.005 µg/mL–33.4 µg/mL, 0.05 µg/mL–334 µg/mL, and 1.37 µg/mL–334 µg/mL for G1, G2, ABT, and 4-MP, respectively.

### Quantification of purines in human and mouse blood and plasma

The analytical reference standards of guanosine, guanine, xanthine, inosine, and hypoxanthine and the internal standards for the assay (guanosine-13C,15N2, guanine-15N5, xanthine-13C,15N2, inosine-13C2,15N, hypoxanthine-13C2,15N) were all purchased from Toronto Research Chemicals (ON, Canada). Plasma concentration of purines in human, CD-1, and A/J mice were determined by a HPLC-MS/MS method. The plasma samples were prepared for analysis by placing a 25 µL aliquot into a 96-well plate followed by the addition of 200 µL of acetonitrile containing an internal standard mixture. The samples were vortexed and centrifuged at 4,000 rpm for 10 min at 4°C; 100 µL of the supernatant was diluted with 50 µL water and ready for analysis. A Shimadzu Nexera UPLC system coupled to a QTRAP 5500 SCIEX in positive ion mode (SCIEX, Foster City, CA) was used for sample analysis. The mobile phases were 10 mM ammonium acetate in water (A) and 10 mM ammonium acetate in acetonitrile:water, 95:5 (B). The gradient method used a total flow rate of 0.6 mL/min to separate samples that were injected onto a Sigma Ascentis Express OH5 (50 × 2.1 mm, 2.7 µm) analytical column for a total run time of 6.0 min. Data were acquired using MRM in positive ion electrospray mode. A duplicate set of calibration curves was prepared using analyte-free surrogate matrix (0.1% bovine serum albumin (BSA) in PBS) with known concentrations of analytes at eight concentration levels and included in each HPLC-MS/MS run of the plasma samples. The peak area ratio of each analyte to its corresponding IS vs concentration of the analyte was fit with a weighted linear curve to determine the unknown analyte concentrations in the plasma samples.

#### Expression and purification

The *A. baumannii* GuaB with CBS domain deletion (M1-G488_ΔM93-R201) was cloned into a modified pET52b vector to produce a fusion protein with an N-terminal His_6_ tag. The plasmid was transformed into BL21 (DE3) Gold cells. Cells were grown at 37°C in Luria broth (LB) medium to an optical density (OD)_600_ of ∼0.8 before induction with 0.5 mM isopropyl beta-D-1-thiogalactopyranoside (IPTG) at 16°C overnight. The cells were harvested by centrifugation, resuspended in lysis buffer (50 mM TrisCl pH 8.0, 300 mM NaCl, and 0.5 mM tris(2-carboxyethyl)phosphine (TCEP), and passed through a microfluidizer. The lysate, clarified by centrifugation, was purified using affinity chromatography on a 5 mL HisTrap FF column (GE Healthcare) pre-equilibrated in buffer A (50 mM TrisCl pH 8.0, 300 mM NaCl, 0.5 mM TCEP, and 10 mM imidazole). A linear concentration gradient of imidazole was applied using buffer B (50 mM TrisCl pH 8.0, 300 mM NaCl, 0.5 mM TCEP and 250 mM imidazole) to elute the protein. The GuaBΔCBS protein was further purified by size-exclusion chromatography using a Superdex200 26/60 column (GE Healthcare) equilibrated with 20 mM TrisCl, pH 7.5, 300 mM NaCl, and 0.5 mM TCEP. Fractions containing the protein were pooled and concentrated to 21 mg/mL for crystallization.The *S. aureus* GuaBΔCBS (W2-F488_ΔE92-R219) and *E. coli* GuaBΔCBS (L2-S488_ΔE90-R217) were cloned, expressed, and purified using the same protocol described above.

#### Crystallization

All GuaB crystals were crystallized in the presence of IMP at room temperature by vapor diffusion method. *A. baumannii* GuaBΔCBS protein (21 mg/mL) was incubated with 5 mM IMP before mixing 1:1 with reservoir solution (0.1 M HEPES, pH 7.5, 20% isopropanol, and 10% PEG4000). Protein complexes bound to small-molecule inhibitors were obtained by soaking the crystals one to two nights in artificial mother liquor with 5 mM of respective compound and 5 mM IMP, then dipping into cryo-protectant solution consisted of 0.1 M HEPES pH 7.5, 10% PEG 4000, 20% ethylene glycol, 2.5 mM IMP, and 2.5 mM compound, before flash frozen in liquid nitrogen. Data sets were collected at APS_24IDC and SSRL12-2. *S. aureus* GuaBΔCBS (27 mg/mL) were crystallized in the presence of 5 mM IMP under the following condition: 0.1 M bicine pH 9.0, 0.1 M NaCl, and 30% PEG550 monomethyl ether (MME). Complex structures were obtained by soaking the crystals overnight in artificial mother liquor with 5 mM of compound and 5 mM IMP. Crystals were cryo-protected with 10% ethylene glycol, 2.5 mM IMP, and 2.5 mM compound in mother liquor, and flash frozen in liquid nitrogen. Data sets were collected at APS_24IDC. *E. coli* GuaBΔCBS (28 mg/mL) with 5 mM of IMP were crystallized in 1 + 1 µL drops with 0.1 M bicine pH 9.0 and 20% PEG 6000. Crystals were soaked overnight in well solution + 5 mM IMP and 0.5 mM–5 mM compound, cryo-protected with mother liquor containing 20% ethylene glycol, 2.5 mM IMP, and 0.25 mM–2.5 mM compound, and flash frozen in liquid nitrogen. Data sets were collected at APS_24IDC.

#### Structure determination

Structures of each species’ GuaB were determined by molecular replacement (MR) using our prior structures, those originally derived from a MR search with published GuaB CBS-deletion coordinates (e.g., *V. cholera* PDB 4IX2). Both the *S. aureus* and *E. coli* GuaB proteins crystallized with one monomer per asu while the fourfold operator of the I4 symmetry space group reproduced the frequently observed GuaB tetramer arrangement in which the NAD binding region and where our inhibitors are located occurs at the interface between adjacent protomers. The *A. baumannii* GuaB consistently crystallized in space group P2_1_ with eight molecules per asu, forming an octamer of two inverted tetramers interdigitating the flexible finger motifs while amino- and carboxy-terminal segments are mutually engaged in strand-swapped interactions with their neighboring protomers around the central axis of the tetramer layer. As with most GuaB structures, the “flap” region above the NAD+ binding pocket is disordered in all cases with ~12–20 residues not discernible. Structures were manually rebuilt using COOT (49) and refined with Phenix (50) and Buster (46). Molecular graphics were prepared with PyMOL [Schrödinger, LLC, (2010) The PyMOL Molecular Graphics System, version 1.5]. Structural models have been refined to good statistics (Table S2) and deposited with structure factors at the PDB under codes (*A.b*. G1: 9AUV, *S.a*. G1: 9AUY, *E.c*. G1: 9AV1, *A.b*. G2: 9AUW, *S.a*. G2: 9AUZ, *E.c*. G2: 9AV2, *A.b*. G8: 9AUX, *S.a*. G8: 9AV0, *E.c*. G8: 9AV3).

#### WaterMap analysis

The crystal structures of three compounds complexed with GuaB *E. coli*, GuaB *S. aureus*, and GuaB *A. baumannii* were first prepared using 2023-1 release of Maestro (Schrodinger). Protein Preparation Wizard (51) was then used to cap the N- and C-terminal with acetyl and N-methyl amide groups, respectively. Protonation states for histidine (HIS), glutamic acid (GLU), and aspartic acid (ASP) and conformational flips of HIS, asparagine (ASN), and glutamine (GLN) side chains were optimized using PROPKA (51) at pH of 7.4. Restrained minimization was carried out using the OPLS4 force field (52), with heavy atoms converged to a root mean square deviation of 0.3 Å. With the bound ligand removed from the binding site of the prepared protein structure, WaterMap (53, 54) simulations were performed using the default simulation parameters (TIP4P solvent model at 300 K, one atmospheric pressure, and 2 ns of simulation time). The crystal waters were kept as a part of explicit solvent. The binding site was defined using the coordinates of the co-crystallized ligand. The truncate protein option was left unchecked for this study. After the simulation is done, the inhomogeneous solvation theory was then applied to determine the enthalpies and entropies of predicted waters.

##### Chemistry experimentals

###### G1

(2S)-N-(6-chloro-3-pyridyl)-2-(2,4-dihydro-1H-pyrano[3,4-c]quinolin-9 ylamino)propanamide

**Scheme** (Fig. S7)

Step 1: 4-methoxybut-1-yne

To a mixture of but-3-yn-1-ol (666 g, 9.5 mol, 720 mL) in H_2_O (1.6 L) was added dropwise a solution of NaOH (660 g, 16.4 mol) in H_2_O (1.60 L) at 10°C. Then Me_2_SO_4_ (830 g, 6.6 mol, 620 mL) was added dropwise at 10°C. The reaction mixture was heated to 110°C and stirred for 15 h. After cooling to 25°C, this reaction batch was combined with two other batches for workup. The organic phase was separated, dried by MgSO_4_ overnight, and then distilled (45°C–85°C, 1 atm) to give 4-methoxybut-1-yne (INT-1, 1.09 kg, crude product) as a light yellow oil, which was used in the next step directly. ^1^H NMR (400 MHz, CDCl_3_) δ 1.99–2.00 (t, *J* = 2.4 Hz, 1H), 2.44–2.48 (td, *J* = 2.8, 6.8 Hz, 2H), 3.39 (s, 3H), 3.50–3.53 (t, *J* = 6.4 Hz, 2H). ^13^C NMR (400MHz, CDCl_3_) δ 81.27, 70.57, 69.23, 58.69, 19.66.

Step 2: methoxypent-2-yn-1-ol

To a mixture of 4-methoxybut-1-yne (280 g, 3.3 mol) in tetrahydrofuran (THF) (1.40 L) at −70°C was added n-BuLi (2.50 M, 1.60 L) dropwise at −70°C under N_2_. After stirring at −70°C for 1 h, formaldehyde (150 g, 4.0 mol) was added in one portion at −70°C under N_2_. The mixture was gradually warmed to 0°C and stirred at 0°C for 2 h. Thin-layer chromatography (TLC) (petroleum ether/ethyl acetate = 1/1, starting material: R_f_ = 0.60, product: Rf = 0.45) showed starting material was consumed completely and new spots were detected. The reaction mixture was quenched with sat. NH_4_Cl solution (3.60 L) then combined with two other same scale batches together for workup. The aqueous phase was adjusted to pH = 4 by conc. HCl solution and extracted with dichloromethane (2.00 L × 3). The combined organic phases were dried over Na_2_SO_4_, filtered, and concentrated under reduced pressure to give a yellow oil. The residue was purified by column chromatography (silica gel, petroleum ether/ethyl acetate = 100/1 to 3/1) to give methoxypent-2-yn-1-ol (INT-2, 495 g, 43.4% yield) as a yellow oil.

^1^H NMR (400 MHz, CDCl_3_) δ 2.15 (s, 1H), 2.48–2.52 (m, 2H), 3.37 (s, 3H), 3.48–3.52 (t, *J* = 6.8 Hz, 2H), 4.24 (s, 2H). ^13^C NMR (400 MHz, CDCl_3_) δ 82.92, 79.51, 70.69, 58.66, 51.22, 19.94.

Step 3: 1-bromo-5-methoxypent-2-yne

To a mixture of methoxypent-2-yn-1-ol (550 g, 4.8 mol), pyridine (50 g, 640 mmol, 51 mL) and diethyl ether (860 mL) at 0°C were added PBr_3_ (660 g, 2.4 mol) dropwise at 0°C under N_2_. The mixture was heated to 50°C and stirred at 50°C for 2.5 h. TLC (petroleum ether/ethyl acetate = 1/1, starting material: R_f_ = 0.45, product: R_f_ = 0.65) showed starting material was consumed completely and new spots were detected. The reaction solution was poured into ice water (4 L) slowly. Then the mixture was extracted with dichloromethane (1 L × 3). The combined organic phases were washed with sat. NaHCO_3_ solution (2 L) and brine (2 L), dried over Na_2_SO_4_, filtered, and concentrated under reduced pressure to give crude 1-bromo-5-methoxypent-2-yne (INT-3, 894 g, assuming this step 100% yield, equal to 849 g product) as a yellow oil. The crude product was used directly in Step 6. ^1^H NMR (400 MHz, CDCl_3_) δ 2.51–2.55 (m, 2H), 3.37 (s, 3H), 3.48–3.51 (t, *J* = 6.8 Hz, 3H), 3.92–3.93 (t, *J* = 2.4 Hz, 2H). ^13^C NMR (400 MHz, CDCl_3_) δ 84.68, 76.25, 70.42, 58.74, 20.23, 15.33.

Step 4: tert-butyl (4-bromophenyl)carbamate

A mixture of 4-bromoaniline (500 g, 2.9 mol) and (Boc)_2_O (698 g, 3.2 mol, 730 mL) in THF (3 L) was heated to 80°C and stirred at 80°C for 15 h. TLC (petroleum ether/ethyl acetate = 10/1, starting material R_f_ = 0.25, product R_f_ = 0.5) showed starting material was consumed completely and new spot was detected. This reaction batch was combined with two other same scale batches and then concentrated under reduced pressure to give a gray residue. The residue was triturated with petroleum ether (1 L) and filtered to give tert-butyl (4-bromophenyl)carbamate (INT-4, 2.1 kg, 89% yield) as a gray solid. ^1^H NMR (400 MHz, CDCl_3_) δ 1.534 (s, 9H), 6.51 (s, 1H), 3.36 (s, 3H), 7.27–7.29 (d, *J* = 8.8 Hz, 2H), 7.40–7.42 (d, *J* = 8.80 Hz, 2H). ^13^C NMR (400 MHz, CDCl_3_) δ 152.51, 137.48, 131.89, 120.05, 115.43, 80.91, 28.31. high-resolution mass spectrometry (HRMS): calcd for C_11_H_15_BrNO_2_*m/z* (M + H, minus t-butyl carmate fragment) 268.0337, 270.0317; found 268.0315, 270.0297.

Step 5: tert-butyl (4-bromophenyl)(5-methoxypent-2-yn-1-yl)carbamate

To a solution of tert-butyl (4-bromophenyl)carbamate (460 g, 1.7 mol) in THF (2 L) was added NaH (81 g, 2.0 mol, 60% purity) in portions at 0°C under N_2_. The mixture was stirred at 0°C for 0.5 h and 1-bromo-5-methoxypent-2-yne (300 g, 1.7 mol) in THF (300 mL) was added dropwise at 0°C. The mixture was then gradually warmed to 25°C and stirred at 25°C for 5 h. TLC (petroleum ether/ethyl acetate = 10/1, starting material: R_f_ = 0.65, product: R_f_ = 0.45) showed starting material was consumed completely and new spots were detected. This reaction batch was combined with two other same scale batches and then quenched with sat. NH_4_Cl solution (3 L) dropwise at 0°C. The mixture was extracted with dichloromethane (1.5 L × 3). The combined organic phases were washed with brine (2 L), dried over Na_2_SO_4_, filtered, and concentrated under reduced pressure to give tert-tert-butyl (4-bromophenyl)(5-methoxypent-2-yn-1-yl)carbamate (INT-5, 1.86 kg, crude product, assuming this step 100% yield, equal to 1.86 kg product) as a brown oil. ^1^H NMR (400 MHz, CDCl_3_) δ 1.47(s, 9H), 2.44–2.48 (m, 2H), 3.37 (s, 3H), 3.44–3.48 (t, *J* = 7.2 Hz, 2H), 4.34–4.35 (t, *J* = 2.0 Hz, 2H), 7.22–7.24 (d, *J* = 8.8 Hz, 2H), 7.44–7.49 (d, *J* = 8.4 Hz, 2H). ^13^C NMR (400 MHz, CDCl_3_) δ 153.82, 141.29, 131.64, 128.00, 120.04, 81.18, 76.10, 70.77, 58.65, 39.90, 28.27, 19.96. HRMS: calcd for C_17_H_23_BrNO_3_*m/z* (M + H, minus t-butyl fragment) 312.0235, 314.0215; found 312.0217, 314.0197.

Step 6: 4-bromo-*N*-(5-methoxypent-2-yn-1-yl)aniline

A mixture of tert-butyl (4-bromophenyl)(5-methoxypent-2-yn-1-yl)carbamate (930 g, 2.5 mol) in HCl/methanol (4 M, 2.2 L) was stirred at 25°C for 15 h. TLC (petroleum ether/ethyl acetate = 5/1, starting material: R_f_ = 0.60, product: R_f_ = 0.45) showed starting material was consumed completely and desired product was detected. This reaction batch was combined with another same scale batch and then concentrated under reduced pressure to give 4-bromo-*N*-(5-methoxypent-2-yn-1-yl)aniline (INT-6, 1.4 kg, crude product, assume this step 100% yield, equal to 1.35 kg product) as a brown oil. ^1^H NMR (400 MHz, CDCl_3_) δ 2.36–2.39 (t, *J* = 6.8 Hz, 2H), 3.28 (s, 3H), 3.37–3.40 (t, *J* = 6.8, 2H), 3.80 (s, 2H), 6.46–6.48 (d, *J* = 8.80 Hz, 2H), 7.19–7.21 (d, *J* = 8.4 H z, 2H). ^13^C NMR (400 MHz, CDCl_3_) δ 146.21, 131.88, 115.06, 110.01, 80.67, 77.01, 70.78, 58.70, 34.06, 19.95. HRMS: calcd for C_12_H_15_BrNO *m/z* (M + H) 268.0337, 270.0317; found 268.0315, 270.0297.

Step 7: 6-bromo-3-iodo-4-(2-methoxyethyl)quinoline

To mixture of I_2_ (1.41 kg, 5.54 mol) and NaHCO_3_ (564 g, 6.7 mol) in MeCN (1.6 L) at 0°C under N_2_ was added dropwise a solution of 4-bromo-*N*-(5-methoxypent-2-yn-1-yl)aniline (450 g, 1.7 mol) in MeCN (1.6 L). The mixture was then heated to 80°C and stirred for 5 h. TLC (petroleum ether/ethyl acetate = 5/1, starting material R_f_ = 0.45, product R_f_ = 0.7) showed starting material was consumed and desired product was detected. After cooling, the mixture was combined with two other same scale batches, diluted with dichloromethane (4 L) and quenched with sat Na_2_SO_3_ solution (15 L). The organic phase was separated, dried over Na_2_SO_4_, filtered, and concentrated under reduced pressure to give a dark residue. The residue was purified by column chromatography (SiO_2_, petroleum ether/tetrahydrofuran = 100/1 to 5/1) to give 6-bromo-3-iodo-4-(2-methoxyethyl)quinoline (INT-7, 515 g, 23% yield, 90% purity) as a yellow solid. ^1^H NMR (400 MHz, CDCl_3_) δ 3.40 (s, 3H), 3.50–3.53 (t, *J* = 7.6 Hz, 3H), 3.64–3.67 (t, *J* = 6.8 Hz, 2H), 7.78–7.81 (dd *J* = 2.4 Hz, 1H), 7.92–7.95 (d, *J* = 9.20 Hz, 1H), 8.28–8.29 (d, *J* = 2.0 Hz, 1H), 9.11 (s, 1H). ^13^C NMR 400 MHz, CDCl_3_) δ 157.41, 146.49, 145.97, 133.09, 131.78, 129.98, 126.75, 121.69, 99.1, 70.52, 58.97, 37.17. HRMS: calcd for C1_2_H_12_BrINO *m/z* (M + H) 391.9147, 393.9125; found 391.9143, 393.9125

Step 8: 6-bromo-4-(2-methoxyethyl)quinoline-3-carbonitrile

A mixture of Pd(PPh_3_)_4_ (69 g, 59 mmol), Zn(CN)_2_ (35 g, 300 mmol) and 6-bromo-3-iodo-4-(2-methoxyethyl)quinoline (230 g, 590 mmol) in N-methyl-2-pyrrolidone (NMP) (1.6L) was heated to 90°C and stirred for 15 h. TLC (petroleum ether/ethyl acetate = 5/1, starting material R_f_ = 0.7, product R_f_ = 0.4) showed starting material was consumed completely. The crude reaction mixture was combined with another similar scale batch for workup. After addition of water (4 L), the mixture was filtered and the filtrate was extracted with methyl tert-butyl ether (MTBE) (1.5L × 3). The combined organic phases were washed with brine (2 L), dried over Na_2_SO_4_, filtered, and concentrated under reduced pressure to give a brown residue. The crude product was triturated with methanol at 25°C for 2 h to give 6-bromo-4-(2-methoxyethyl)quinoline-3-carbonitrile (INT-8, 462 g, crude product, assume this step 100% yield, equal to 382 g product) as a yellow solid. Due to the poor solubility of INT-8, the crude material was carried forward to the next step without further purification. ^1^H NMR (400 MHz, CDCl_3_) δ 3.27 (s, 3H), 3.47–3.51 (t, *J* = 6.4 Hz, 2H), 3.70–3.74 (t, *J* = 6.4 Hz, 2H), 7.84–7.87 (dd, *J* = 2.4 Hz, 1H), 7.96–7.98 (d, *J* = 8.8 Hz, 1H), 8.26 (d, *J* = 2.0 Hz, 1H), 8.91 (s, 1H). ^13^C NMR (400 MHz, CDCl_3_) δ 151.22, 150.22, 147.42, 135.52, 132.13, 127.52, 127.12, 122.59, 116.49, 108.47, 71.35, 59.02, 31.67.

Step 9: 9-bromo-1,2-dihydro-4H-pyrano[3,4-c]quinolin-4-one

A mixture of 6-bromo-4-(2-methoxyethyl)quinoline-3-carbonitrile (190 g, 660 mmol) in HCl (12 M, 5.8 L) was heated to 120°C and stirred for 15 h. TLC (petroleum ether/ethyl acetate = 5/1, starting material R_f_ = 0.7, product R_f_ = 0.45) showed starting material was consumed completely. The reaction mixture was cooled to 30°C, stirred at 30°C for 1 h, and combined with other same scale batch for workup. The reaction mixture was filtered and the filtrate was adjusted to pH = 8 by conc. NH_4_OH at 0°C. The suspension was filtered and the filter cake was dried under reduced pressure to give 9-bromo-1,2-dihydro-4H-pyrano[3,4 c]quinolin-4-one (INT-9, 204 g, 56% yield) as a yellow solid, which was used for the next step directly. ^1^H NMR (400 MHz, CDCl_3_) δ 3.42–3.45 (t, *J* = 6.0 Hz, 2H), 4.71–4.74 (t, *J* = 6.0 Hz, 2H), 7.93–7.96 (dd, *J* = 2.0 Hz, 1H), 8.06–8.08 (d, *J* = 9.2 Hz, 1H), 8.16–8.17 (d, *J* = 4.0 Hz, 1H), 9.46 (s, 1H). ^13^C NMR (400 MHz, CDCl_3_) δ 163.36, 150.29, 148.04, 146.32, 135.58, 132.25, 126.59, 125.61, 122.28, 118.50, 66.18, 23.58. HRMS: calcd for C_12_H_9_BrNO *m/z* (M + H) 277.9817, 279.9796; found 277.9802, 279.9781

Step 10: 2-(6-bromo-3-(hydroxymethyl)quinolin-4-yl)ethan-1-ol

To a solution of 9-bromo-1,2-dihydro-4H-pyrano[3,4-c]quinolin-4-one (184 g, 660 mmol) in THF (1.3 L) at 0°C was added LiAlH_4_ (23 g, 600 mmol) in portions at 0°C under N_2_. The reaction was stirred at 0°C for 2 h. The reaction mixture was quenched with methanol (120 mL) dropwise at 0°C and concentrated under reduced pressure to give a yellow residue. The residue was purified by chromatography on a silica gel eluting with dichloromethane/methanol (from 100/1 to 20/1) to give 2-(6-bromo-3-(hydroxymethyl)quinolin-4-yl)ethan-1-ol (INT-10, 130 g, 70% yield) as a yellow solid. ^1^H NMR (400 MHz, CDCl_3_) δ 3.26–3.30 (t, *J* = 6.8 Hz, 2H), 3.65–3.70 (dd, *J* = 6.8 Hz, 2H), 4.76–4.77 (d, *J* = 5.60 Hz, 2H), 4.92–4.94 (t, *J* = 5.6 Hz, 1H), 5.36–5.39 (t, *J* = 5.6 Hz, 1H), 7.82–7.84 (dd, *J* = 2.0 Hz, 1H), 7.92–7.94 (d, *J* = 8.80 Hz, 1H), 8.38–8.39 (d, *J* = 1.60 Hz, 1H), 8.89 (s, 1H). ^13^C NMR (400 MHz, CDCl_3_) δ 151.58), 145.80, 141.85, 133.76, 131.69, 128.50, 126.54, 119.77, 60.89, 59.06, 59.06, 30.58. LC-MS M/Z (M + H) 283.0. HRMS: calcd for C_12_H1_3_BrNO_2_*m/z* (M + H) 282.0130, 284.0109; found 282.0148, 284.0129

Step 11: 9-bromo-1,4-dihydro-2H-pyrano[3,4-c]quinoline

A mixture of 2-(6-bromo-3-(hydroxymethyl)quinolin-4-yl)ethan-1-ol (110 g, 390 mmol) and H_3_PO_4_ (2.3 kg, 23.6 mol, 1.4 L) was stirred and heated to 140°C for 20 h. After cooling, the mixture was poured into ice water (2 L) and adjusted to pH = 8 by conc. NH_4_OH solution. The suspension was filtered and the filter cake was dried under vacuum to give a gray solid. The crude product was triturated with water (1 L) at 25°C for 2 h. The mixture was filtered and the filter cake was triturated with methanol (200 mL) at 25°C for 2 h to give 9-bromo-1,4-dihydro-2H-pyrano[3,4 c]quinoline (INT-11, 102 g, 99% yield, 98.6% purity) as a gray solid. ^1^H NMR (400 MHz, CDCl_3_) δ 3.10–3.13 (t, *J* = 5.6 Hz, 2H) 4.12–4.15 (t, *J* = 6.0 Hz, 2H), 4.93 (s, 2H), 7.74–7.76 (dd, *J* = 2.0 Hz, 1H) 7.94–7.96 (d, *J* = 9.20 Hz, 1H), 8.02–8.03 (d, *J* = 2.00 Hz, 1H), 8.56 (s, 1H). ^13^C NMR (400 MHz, CDCl_3_) δ 148.11, 145.40, 137.48, 132.16, 131.84, 128.59, 128.01, 124.98, 121.10, 66.03, 64.43, 24.32. LC-MS M/Z (M + H) 264.0. HRMS: calcd for C_12_H_11_BrNO *m/z* (M + H) 264.0024, 266.0004; found 264.0033, 266.0014.

Step 12: tert-butyl N-[(1S)-2-[(6-chloro-3-pyridyl)amino]-1-methyl-2-oxo-ethyl]carbamate

To a mixture of (2S)-2-[(tert-butoxycarbonyl)amino]propanoic acid (4.42g, 23.34mmol) in N,N-dimethylformamide (DMF) (20mL) was added N,N-diisopropylethylamine (13.85 mL, 77.78 mmol), hexafluorophosphate azabenzotriazole tetramethyl uronium (HATU) (11. 83mg, 31.11mmol), and 6-chloro-3-pyridinamine (2.0 g, 15.56 mmol). The resulting mixture was stirred at 20°C under nitrogen atmosphere for 2 h. After that, the reaction mixture was diluted with water (50 mL), and extracted with EtOAc (100 mL). The organic phase was washed with brine (20 mL), dried over Na_2_SO_4_, and concentrated to dryness *in vacuo*. The residue was purified by flash column chromatography eluting with 0 to 50% EtOAc in petroleum ether to afford tert-butyl N-[(1S)-2-[(6-chloro-3-pyridyl)amino]-1-methyl-2-oxo-ethyl]carbamate (INT-12, 4.0 g, 86% yield) as a brown solid. ^1^H NMR (400 MHz, DMSO-*d6*) δ 10.32 (s, 1H), 8.60 (d, *J* = 2.8 Hz, 1H), 8.10 (dd, *J* = 2.4, 8.8 Hz, 1H), 7.47 (d, *J* = 8.4 Hz, 1H), 7.19 (d, *J* = 6.8 Hz, 1H), 4.12–4.08 (m, 1H), 1.36 (s, 9H), 1.26 (d, *J* = 7.6 Hz, 1H). ^13^C NMR (101 MHz, DMSO-*d*_6_) δ 173.07, 155.71, 143.98, 140.85, 135.82, 130.26, 124.66, 78.62, 50.94, 28.67, 18.16. HRMS: calcd for C_13_H_19_ClN_3_O_3_ *m/z* (M + H) 300.1037, found 300.1112.

Step 13: (2S)-2-amino-N-(6-chloro-3-pyridyl)propanamide hydrochloride

To a mixture of tert-butyl N-[(1S)−2-[(6-chloro-3-pyridyl)amino]-1-methyl-2-oxo-ethyl]carbamate (4.0 g, 13.34 mmol) in EtOAc (40 mL) was added 4M HCl in EtOAc (16.7 mL). The resulting mixture was stirred at 25°C for 2 h. After that, the reaction mixture was concentrated to afford (2S)-2-amino-N-(6-chloro-3-pyridyl)propanamide hydrochloride (INT-13, 2.2 g, 70% yield) as a yellow solid. ^1^H NMR (400 MHz, DMSO-*d6*) δ 11.63 (s, 1H), 8.73 (d, *J* = 2.4 Hz, 1H), 8.47 (br, 3H), 8.14 (dd, *J* = 2.8, 8.8 Hz, 1H), 7.49 (d, *J* = 8.4 Hz, 1H), 4.20–4.04 (m, 1H), 1.47 (d, *J* = 7.2 Hz, 3H). ^13^C NMR (101 MHz, DMSO-*d*_6_) δ 170.15, 144.69, 141.04, 135.23, 130.51, 124.87, 111.02, 49.66, 17.90. HRMS: calcd for C_8_H_11_ClN_3_O *m/z* (M + H) 200.0512, found 200.0588.

Step 14: (2S)-N-(6-chloro-3-pyridyl)-2-(2,4-dihydro-1H-pyrano[3,4-c]quinolin-9-ylamino)propan-amide, (G1)

Two parallel reactions were set up: a mixture of 9-bromo-1,4-dihydro-2H-pyrano[3,4-c]quinoline (400 mg, 1.51 mmol), (2S)-2-amino-N-(6-chloro-3-pyridyl)propanamide hydrochloride (429 mg, 1.82 mmol), cesium carbonate (1.23 g, 3.79 mmol), copper (I) iodide (28.8 mg, 0.15 mmol), and 2-isobutyrylcyclohexanone (51 mg, 0.30 mmol) was degassed for three times under nitrogen atmosphere. Then DMSO (8 mL) was added and the resulting mixture was heated at 80°C for 12 h under nitrogen atmosphere. After that, the two parallel reactions were combined, cooled down to room temperature, diluted with EtOAc (100 mL) and water (50 mL), and fitered through diatomite. The organic layer was separated and the aqueous phase was extracted with EtOAc (20 mL × 2). The combined organic phases were washed with brine (10 mL × 2) and concentrated. The residue was purified by flash column chromatography on silica gel eluting with 10% to 30% EtOH in dichloromethane to provide a partially purified product, which was re-purified by flash column chromatography on silica gel eluting with 55% (EtOAc:ethanol = 3:1) in petroleum ether to afford crude (2S)-N-(6-chloro-3-pyridyl)-2-(2,4-dihydro-1H-pyrano[3,4 c]quinolin-9-ylamino)propan-amide 729 mg, 72% ee, 63% yield) as a yellow oil. Further separation by supercritical fluid chromatography (SFC) (Column: YMC CHIRAL Amylose-C (250 mm * 30 mm, 10umk, 0.1%NH_3_.H_2_O EtOH, 55% EtOH) afforded the enantiomerically pure (2S)-N-(6-chloro-3-pyridyl)-2-(2,4-dihydro-1H-pyrano[3,4 c]quinolin-9-ylamino)propan-amide (G1, 500.2 mg, 99% ee, 69% yield) as a brown solid. ^1^H NMR (400 MHz, DMSO-*d6*) δ 10.60 (s, 1H), 8.62 (d, *J* = 3.2 Hz, 1H), 8.23 (s, 1H), 8.10 (dd, *J* = 8.8, 2.8 Hz, 1H), 7.71 (d, *J* = 8.8 Hz, 1H), 7.45 (d, *J* = 8.8 Hz, 1H), 7.24 (dd, *J* = 9.2, 2.0 Hz, 1H), 6.65 (d, *J* = 2.4 Hz, 1H), 6.61 (d, *J* = 7.2 Hz, 1H), 4.77 (s, 2H), 4.26–4.22 (m, 1H), 4.03–3.97 (m, 2H), 2.98–2.94 (m, 1H), 2.77–2.72 (m, 1H), 1.50 (d, *J* = 6.8 Hz, 3H), LC-MS M/Z (M + H) 383.1. ^13^C NMR (101 MHz, DMSO-*d*_6_) δ 174.14, 146.51, 144.30, 143.40, 141.22, 141.18, 135.52, 135.23, 130.71, 130.64, 128.48, 128.31, 124.68, 120.79, 98.54, 65.77, 64.39, 53.60, 24.43, 18.86. LC-MS M/Z (M + H) 383.0. HRMS: calcd for C_20_H_20_ClN_4_O_2_ *m/z* (M + H) 383.1197, found 383.1263.

###### G2

(2S)-N-[4-chloro-3-(dimethylamino)phenyl]-2-[[3-(hydroxymethyl)-6-quinolyl]amino]propanamide

**Scheme** (Fig. S8)

Step 1: (6-bromoquinolin-3-yl)methanol

To a mixture of methyl 6-bromoquinoline-3-carboxylate (55.0 g, 206.7 mmol) in dichloromethane (1L) was added dropwise a solution of DIBAL-H (516.74 mL, 516.74 mmol, 1M in toluene) at 0°C. After further stirring for 2 h, the mixture was quenched slowly with H_2_O (21 mL) at 0°C, followed by 15 mL of 15% NaOH solution and 52 mL of water. The mixture was stirred at room temperature for 10 min, followed by the addition of Na_2_SO_4_ (50 g) and stirred for another 10 min. The mixture was then filtered and concentrated *in vacuo*. The residue was purified by flash chromatography on silica gel eluting with petroleum ether/EtOAc (from 5:1 to 1:1), CH_2_Cl_2_/MeOH (10:1) to give (6-bromoquinolin-3-yl)methanol (INT-14, 17 g, 34.5%) as a red oil. ^1^H NMR (400 MHz, DMSO-*d*_6_) δ 8.89 (d, *J* = 2.1 Hz, 1H), 8.29 (d, *J* = 2.3 Hz, 1H), 8.23 (dt, *J* = 2.1, 1.0 Hz, 1H), 7.95 (d, *J* = 8.9 Hz, 1H), 7.83 (dd, *J* = 8.9, 2.3 Hz, 1H), 5.55–5.48 (m, 1H), 4.73 (d, *J* = 4.6 Hz, 2H). ^13^C NMR (101 MHz, DMSO-*d*_6_) δ 151.40, 136.87, 132.45, 132.16, 131.39, 130.32, 129.45, 120.06, 61.20. LC-MS M/Z (M + H) 238. HRMS: calcd for C_11_H_8_BrNO *m/z* (M + H) 237.9789; found 237.9864.

Step 2: 6-bromo-3-(tetrahydropyran-2-yloxymethyl)quinoline

To a solution of (6-bromoquinolin-3-yl)methanol (22.0 g, 92.41 mmol) in CH_2_Cl_2_ (200 mL) was added 3,4-dihydro-2H-pyran (38.87 g, 462.03 mmol) and pyridinium p-toluenesulfonate (PPTS) (4.64g, 18.48mmol) at 10°C ~ 20°C. Then the mixture was stirred at 40°C for 15 h and then concentrated *in vacuo*. The residue was purified on silica gel column eluting with EtOAc/petroleum ether (20% ~50% EtOAc in petroleum ether) to afford 6-bromo-3-(tetrahydropyran-2-yloxymethyl)quinoline (INT-15, 28 g, 94%) as a white solid. ^1^H NMR (400 MHz, CDCl_3_) δ 8.84 (d, *J* = 1.6 Hz, 1 H) 7.98 (s, 1 H) 7.78–7.95 (m, 2 H) 7.69 (dd,*J* = 9.2, 2.0 Hz, 1 H) 4.91 (d, *J* = 12.4 Hz, 1 H) 4.70 (t, *J* = 3.2 Hz, 1 H) 4.63 (d, *J* = 12.4 Hz, 1 H) 3.74–3.94 (m, 1 H) 3.26–3.61 (m, 1 H) 1.45–1.94 (m, 6 H). ^13^C NMR (101 MHz, DMSO-*d*_6_) δ 151.78, 146.08, 133.66, 132.96, 132.86, 131.41, 130.47, 129.3, 120.21, 98.15, 66.29, 61.90, 30.58, 25.45, 19.46. HRMS: calcd for C_15_H_17_BrNO_2_ *m/z* (M + H) 322.0364; found 322.0438.

Step 3: (2S)-2-[[3-(tetrahydropyran-2-yloxymethyl)-6-quinolyl]amino] propanoic acid

A mixture of 6-bromo-3-(tetrahydropyran-2-yloxymethyl)quinoline (10.0 g, 31.04 mmol), L-alanine (41.48 g, 46.55 mmol), cesium carbonate (15.17 g, 46.55 mmol), and copper(I) iodide (2.36 g, 12.41 mmol) in DMSO (80 mL) was stirred at 105°C for 12 h. After that, the reaction mixture was cooled down to room temperature, diluted with water (100 mL), and extracted with EtOAc (100 mL × 3). The combined organic layers were washed with water (150 mL × 2) and brine (100 mL), dried over anhydrous Na_2_SO_4_, filtered, and concentrated. The residue was purified by flash chromatography on silica gel eluting with 50% EtOAc in petroleum ether, then 25% mEtOH in dichloromethane to afford (2S)-2-[[3-(tetrahydropyran-2-yloxymethyl)-6-quinolyl]amino] propanoic acid (INT-16, 10 g, 98% yield) as a yellow solid. ^1^H NMR (400 MHz, DMSO-*d*_6_) δ 8.43 (d, *J* = 2.1 Hz, 1H), 7.86 (s, 1H), 7.74–7.57 (m, 1H), 7.30–7.14 (m, 1H), 6.62 (s, 1H), 4.84–4.62 (m, 2H), 4.55 (dd, *J* = 12.2, 1.8 Hz, 1H), 3.81 (td, *J* = 9.2, 7.4, 3.4 Hz, 2H), 3.60–3.46 (m, 1H), 1.81–1.60 (m, 2H), 1.52 (tdd, *J* = 15.7, 7.7, 3.3 Hz, 4H), 1.36 (d, *J* = 6.6 Hz, 3H).

^13^C NMR (101 MHz, DMSO-*d*_6_) δ 146.19, 145.02, 141.38, 131.54, 130.83, 129.38, 128.99, 121.73, 101.45, 97.36, 66.17, 61.29, 52.34, 30.07, 24.91, 18.95, 18.37. LC-MS M/Z (M + H) 330.9. HRMS: calcd for C18H23N2O4*m/z* (M + H) 331.1652; found 331.1654.

Step 4: (2S)-N-[4-chloro-3-(dimethylamino)phenyl]-2-[[3-(tetrahydropyran-2-yloxymethyl)-6-quinolyl]amino]propan-amide

To a solution of (2S)-2-[[3-(tetrahydropyran-2-yloxymethyl)-6-quinolyl]amino] propanoic acid (2.0 g, 6.05 mmol) and HATU (3.22 g, 8.48 mmol) in DMF (15 mL) were added N,N-diisopropylethylamine (4.3 mL, 24.21 mmol) and 4-chloro-N^3^,N^3^-dimethyl-benzene-1,3-diamine (2.07 g, 12.11 mmol). The resulting mixture was stirred at 20°C for 3 h. After that, the reaction mixture was concentrated. The residue was purified by flash column chromatography eluting with 0 to 5% mEtOH in dichloromethane to afford (2S)-N-[4-chloro-3-(dimethylamino)phenyl]-2-[[3-(tetrahydropyran-2-yloxymethyl)-6-quinolyl]amino]propan-amide (INT-17, 2.2 g, 75% yield) as a yellow solid. ^1^H NMR (400 MHz, DMSO-*d*_6_) δ 10.17 (s, 1H), 8.48 (d, *J* = 2.1 Hz, 1H), 7.90–7.84 (m, 1H), 7.74 (d, *J* = 9.1 Hz, 1H), 7.49 (d, *J* = 2.3 Hz, 1H), 7.36–7.23 (m, 3H), 6.68 (d, *J* = 2.5 Hz, 1H), 6.49 (d, *J* = 7.5 Hz, 1H), 4.78 (d, *J* = 12.3 Hz, 1H), 4.71 (dt, *J* = 4.2, 2.6 Hz, 1H), 4.57 (d, *J* = 12.3 Hz, 1H), 4.16 (p, *J* = 6.9 Hz, 1H), 3.79 (ddd, *J* = 11.4, 8.1, 3.3 Hz, 1H), 3.53–3.42 (m, 1H), 2.69 (s, 6H), 1.84–1.54 (m, 3H), 1.48 (d, *J* = 6.7 Hz, 6H). ^13^C NMR (101 MHz, DMSO-*d*_6_) δ 173.27, 150.67, 146.54, 146.26, 142.29, 139.09, 132.21, 131.66, 130.78, 129.80, 129.62, 122.10, 121.30, 114.39, 111.71, 102.71, 97.95, 66.66, 61.85, 53.59, 43.58, 30.61, 25.45, 19.50, 19.13. LC-MS M/Z (M + H) 483.1. HRMS: calcd for C_26_H_32_ClN_4_O_3_*m/z* (M + H) 483.2085; found 483.2165.

Step 5: (2S)-N-[4-chloro-3-(dimethylamino)phenyl]-2-[[3-(hydroxymethyl)-6-quinolyl]amino]propenamide, (G2)

A solution of (2S)-N-[4-chloro-3-(dimethylamino)phenyl]-2-[[3-(tetrahydropyran-2-yloxymethyl)-6-quinolyl]amino]propan-amide (11.6 g, 20.02 mmol) in 4M HCl in EtOAc (120 mL) was stirred at room temperature for 30 min. The reaction mixture was concentrated under reduced pressure. The residue was purified by reverse HPLC [water (0.1% TFA)-acetonitrile] to afford (2S)-N-[4-chloro-3-(dimethylamino)phenyl]-2-[[3-(hydroxymethyl)-6-quinolyl]-amino]propanamide (4.6 g, 49% ee, 48% yield) as a white solid, which was further purified by SFC [mobile phase: A:CO_2_ B:mEtOH (0.05% DEA) Gradient: hold 5% for 0.2 min, then from 5% to 40% of B in 1.4 min and hold 40% for 1.05 min, then 5% of B for 0.35 min] to afford enantiomeric pure (2S)-N-[4-chloro-3-(dimethylamino)phenyl]-2-[[3-(hydroxymethyl)-6-quinolyl]amino]propanamide (G2, 2.0 g, 99% ee, 44% yield) as a yellow solid. ^1^H NMR (400 MHz, CD_3_OD) δ 8.50 (d, *J* = 2.0 Hz, 1H), 7.97 (s, 1H), 7.78 (d, *J* = 9.2 Hz, 1H), 7.47 (d, *J* = 2.0 Hz, 1H), 7.32 (dd, *J* = 2.4, 9.2 Hz, 1H), 7.27–7.22 (m, 1H), 7.21–7.15 (m, 1H), 6.72 (d, *J* = 2.4 Hz, 1H), 4.72 (s, 2H), 4.12 (q, *J* = 6.8 Hz, 1H), 2.73 (s, 6H), 1.60 (d, *J* = 6.8 Hz, 3H). ^13^C NMR (101 MHz, DMSO-*d*_6_) δ 173.36, 150.68, 146.44, 145.88, 142.07, 139.12, 135.64, 130.80, 130.70, 129.79, 129.74, 121.62, 121.30, 114.36, 111.69, 102.78, 61.48, 53.62, 43.60, 19.13. LC-MS M/Z (M + H) 399.0. HRMS: calcd for C_21_H_24_O_2_N_4_Cl *m/z* (M + H) 399.1582; found 399.1573.

###### G8

(R)-9-[1-[5-(4-chloro-1H-imidazol-2-yl)-3-pyridyl]ethoxy]-2,4-dihydro-1H-pyrano[3,4 c]quinoline

**Scheme** (Fig. S9)

Step 1: 1,4-dihydro-2H-pyrano[3,4-c]quinolin-9-ol

A mixture of 9-bromo-1,4-dihydro-2H-pyrano[3,4-c]quinoline (90 g, 340 mmol, 220 mL), KOH (77 g, 1.4 mol), Pd_2_(dba)_3_ (31 g, 34 mmol), di-tert-butyl-[2-(2,4,6-triisopropylphenyl)phenyl]phosphane (29 g, 68 mmol) in dioxane (900 mL), and H_2_O (900 mL) under N_2_ was heated to 80°C and stirred at 80°C for 15 h. The reaction mixture was cooled to 25°C, diluted with 900 mL of water, and washed with MTBE (500 mL × 3). The aqueous phase was first adjusted to pH = 4 by 2 M HCl solution, then re-adjusted to pH = 7 by sat. NaHCO_3_ solution. The precipitate was collected and triturated with methanol (100 mL) to give 1,4-dihydro-2H-pyrano[3,4 c]quinolin-9-ol (INT-18, 55 g, 80% yield, 98% purity) as a light yellow solid. ^1^H NMR (400 MHz, DMSO-d_6_) δ 2.85–3.04 (m, 2H) 4.01 (t, *J* = 5.6 Hz, 2H) 4.81 (s, 2H) 7.12 (d, *J* = 2.4 Hz, 1H) 7.26 (dd, *J* = 8.8, 2.4 Hz, 1H) 7.84 (d, *J* = 8.8 Hz, 1H) 8.37 (s, 1H) 10.0 (s, 1H). ^13^C NMR (101 MHz, DMSO) δ 155.61, 147.93, 146.18, 145.74, 142.53, 142.33, 138.54, 137.11,

131.63, 130.13, 129.26, 128.73, 127.81, 126.12, 121.60, 115.39, 105.32, 73.38, 65.67, 64.27, 24.37,23.66. HRMS: calcd for C_22_H_21_ClN_4_O_2_ *m/z* (M + H) 407.1275; found 407.1264.

Step 2: 2-[(4-chloroimidazol-1-yl)methoxy]ethyl-trimethylsilane

To a stirred mixture of NaH (7.0 g, 176 mmol) in anhydrous THF (90 mL) was added 4-chloro-1H-imidazole (15 g, 146 mmol) in THF (75 mL) slowly at 5°C. After stirring for 30 min, 2-(trimethylsilyl)ethoxymethyl chloride (39 mL, 220 mmol) was added dropwise and the resulting reaction mixture was stirred at 25°C for another 2 h. The reaction was then quenched with water (10 mL), concentrated, and purified by flash column (0%–30% EtOAc in petroleum ether) to afford 2-[(4-chloroimidazol-1-yl)methoxy]ethyl-trimethylsilane (INT-19, 28 g, 82% yield) as a colorless oil. ^1^H NMR (400 MHz, DMSO-*d6*) δ = 7.79 (d, *J* = 1.6 Hz, 1H), 7.39 (d, *J* = 1.2 Hz, 1H), 5.29 (s, 2H), 3.47 (t, *J* = 8.0 Hz, 2H), 0.84 (t, *J* = 8.0 Hz, 2H), −0.03 (s, 9H). ^13^C NMR (101 MHz, DMSO-*d*_6_) δ 137.91, 116.44, 76.38, 66.42, 17.99,–0.53. HRMS: calcd for C_9_H_18_ClN_2_OSi *m/z* (M + H) 233.0799, found 233.0877.

Step 3: 2-[(2-bromo-4-chloro-imidazol-1-yl)methoxy]ethyl-trimethylsilane

To a stirred solution of 2-[(4-chloroimidazol-1-yl)methoxy]ethyl-trimethylsilane (15 g, 64 mmol) in anhydrous THF (150 mL) was added n-BuLi (31 mL, 77 mmol) dropwise at −76°C under nitrogen atmosphere. The resulting mixture was stirred at −76°C for 30 min and N-bromosuccinimide (NBS) (12.6g, 70.9mmol) in anhydrous THF (180mL) was added dropwise. The resulting mixture was stirred for 30 min, then slowly warmed up to 25°C and stirred for 3 h. Saturated aqueous NH_4_Cl solution (100 mL) was added and the reaction mixture was extracted with EtOAc (2 × 200 mL). The combined organic phases were dried over Na_2_SO_4_, filtered, and concentrated. The residue was purified by flash chromatography on silica gel (0%~6% EtOAc in petroleum ether) to afford 2-[(2-bromo-4-chloro-imidazol-1-yl)methoxy]ethyl-trimethylsilane (INT-20, 6.5 g, 32% yield) as a light yellow oil. ^1^H NMR (400 MHz, CDCl_3_) δ 7.01 (s, 1H), 5.21 (s, 2H), 3.57–3.51 (m, 2H), 0.94–0.89 (m, 2H), 0.01 (s, 9H). ^13^C NMR (101 MHz, DMSO-*d*_6_) δ 128.43, 120.85, 118.86, 76.85, 66.82, 17.96,–0.46. LC-MS M/Z (M + H) 311, 313. HRMS: calcd for C_9_H_17_BrClN_2_OSi *m/z* (M + H) 310.9982, 312.9962, found 310.9982, 312.9958.

Step 4: 1-(5-(4-chloro-1-((2-(trimethylsilyl)ethoxy)methyl)-1H-imidazol-2-yl)pyridin-3-yl) ethanone

A mixture of 1,1´-bis(diphenylphosphino)ferrocene palladium dichloride (94 mg, 0.13 mmol), (5-acetyl-3-pyridyl)boronic acid (233 mg, 1.41 mmol), 2-[(2-bromo-4-chloro-imidazol-1-yl)methoxy]ethyl-trimethylsilane (400 mg, 1.28 mmol), Na_2_CO_3_ (272 mg, 2.57 mmol) in 1,4-dioxane (4 mL), and water (1 mL) was purged with N_2_ for 3 min at 20°C. Then the reaction mixture was heated at 100°C for 6 h under N_2_. After that, the reaction mixture was concentrated *in vacuo*. The residue was purified by preparative TLC (20% EtOAc in petroleum ether) to give 1-(5-(4-chloro-1-((2-(trimethylsilyl)ethoxy)methyl)-1H-imidazol-2-yl)pyridin-3-yl) ethanone (INT-21, 230 mg, 51% yield) as a yellow solid. ^1^H NMR (400 MHz, DMSO-d6) δ 9.19 (d, *J* = 2.0 Hz, 1H), 9.14 (d, *J* = 2.0 Hz, 1H), 8.60 (t, *J* = 2.2 Hz, 1H), 7.75 (s, 1H), 5.40 (s, 2H), 3.61 (t, *J* = 8.2 Hz, 2H), 2.68 (s, 3H), 0.89 (t, *J* = 8.0 Hz, 2H), −0.05 (s, 9H). ^13^C NMR (101 MHz, DMSO-*d*_6_) δ 197.82, 152.95, 150.44, 143.50, 135.55, 132.67, 128.90, 120.77, 76.30, 66.91, 27.98, 18.11, –0.50. HRMS: calcd for C_16_H_23_ClN_3_O_2_Si *m/z* (M + H) 352.1170, found 352.1248.

Step 5: 1-(5-(4-chloro-1-((2-(trimethylsilyl)ethoxy)methyl)-1H-imidazol-2-yl)pyridin-3-yl) ethanol

To a solution of 1-(5-(4-chloro-1-((2-(trimethylsilyl)ethoxy)methyl)-1H-imidazol-2-yl)pyridin-3-yl) ethanone (230 mg, 0.65 mol) in MeOH (5.75 mL) was added sodium borohydride (50 mg, 1.3 mol) at 0°C. The resulting reaction mixture was stirred at 0°C for 2 h. After that, the reaction mixture was concentrated *in vacuo* and diluted with EtOAc (100 mL). The resulting solution was successively washed with water (50 mL × 3) and brine (50 mL), dried over anhydrous Na_2_SO_4_, filtered, and concentrated *in vacuo* to afford 1-(5-(4-chloro-1-((2-(trimethylsilyl)ethoxy)methyl)-1H-imidazol-2-yl)pyridin-3-yl) ethanol (INT-22, 190 mg, 82% yield) as a yellow oil. ^1^H NMR (400 MHz, DMSO-*d6*) δ 8.80 (d, *J* = 2.4 Hz, 1H), 8.63 (d, *J* = 2.0 Hz, 1H), 8.12–8.07 (m, 1H), 7.69 (s, 1H), 5.44 (d, *J* = 4.0 Hz, 1H), 5.35 (s, 2H), 4.88–4.82 (m, 1H), 3.58 (t, *J* = 8.2 Hz, 2H), 1.39 (d, *J* = 6.4 Hz, 3H), 0.87 (t, *J* = 8.2 Hz, 2H), −0.05 (s, 9H). ^13^C NMR (101 MHz, DMSO-*d*_6_) δ 148.57, 147.78, 144.54, 143.17, 133.45, 128.73, 125.73, 120.19, 76.22, 66.83, 66.74, 26.40, 18.08, –0.49. HRMS: calcd for C_16_H_25_ClN_3_O_2_Si *m/z* (M + H) 354.1326, found 354.1406.

Steps 6 and 7: (R)-9-[1-[5-(4-chloro-1H-imidazol-2-yl)-3-pyridyl]ethoxy]-2,4-dihydro-1H-pyrano[3,4-c]quinoline (G8)

A mixture of tri-n-butylphosphine (10.04 mL, 40.69 mmol), N,N,N´,N´-tetramethylazodicarboxamide (7.01 g, 40.69 mmol), 1,4-dihydro-2H-pyrano[3,4 c]quinolin-9-ol (2.25 g, 11.19 mmol), and 1-(5-(4-chloro-1-((2-(trimethylsilyl)ethoxy)methyl)-1H-imidazol-2-yl)pyridin-3-yl) ethanol (3.6 g, 10.17 mmol) in anhydrous toluene (100 mL) was stirred at 25°C for 16 h. After that, the reaction mixture was diluted with EtOAc (100 mL), washed with brine (40 mL × 2), dried over Na_2_SO_4_, filtered, and concentrated. To a stirred solution of the crude INT-23 2-[[4-chloro-2-[5-[1-(2,4-dihydro-1H-pyrano[3,4 c]quinolin-9-yloxy)ethyl]-3-pyridyl]imidazol-1-yl]methoxy]ethyl-trimethylsilane in EtOAc (30 mL) was added 4M HCl in EtOAc (20 mL). The resulting mixture was stirred at 25°C for 3 h and then concentrated *in vacuo* to afford the crude 9-[1-[5-(4-chloro-1H-imidazol-2-yl)-3-pyridyl] ethoxy]-2,4-dihydro-1H-pyrano[3,4 c]quinolone as an off-white solid, which was further purified by chiral SFC separation [DAICEL CHIRALPAK AD (250 mm * 50 mm, 10 µm); condition: 0.1%NH_3_.H_2_O EtOH 40%] to afford desired product as an off-white solid. Further purification by recrystallization (ethyl alcohol) afforded (R)-9-[1-[5-(4-chloro-1H-imidazol-2-yl)-3-pyridyl]ethoxy]-2,4-dihydro-1H-pyrano[3,4 c]quinoline (G8, 1.0 g, 24% yield) as an off-white solid. Exact stereochemistry was confirmed by co-crystal structure of compound G8 bound to GuaB (*A.b*. G8: 9AUX, *S.a*. G8: 9AV0, *E.c*. G8: 9AV3). ^1^H NMR (400 MHz, CD_3_OD) δ 8.93 (d, *J* = 2.0 Hz, 1H), 8.71 (d, *J* = 2.0 Hz, 1H), 8.41 (t, *J* = 2.0 Hz, 1H), 8.36 (s, 1H), 7.89 (d, *J* = 9.2 Hz, 1H), 7.47 (dd, *J* = 2.8, 9.2 Hz, 1H), 7.26 (d, *J* = 2.4 Hz, 1H), 7.21 (s, 1H), 5.85 (q, *J* = 6.4 Hz, 1H), 4.85 (s, 2H), 4.12–4.04 (m, 2H), 3.15–3.08 (m, 1H), 2.94–2.88 (m, 1H), 1.78 (d, *J* = 6.4 Hz, 3H). 13C NMR (101 MHz, DMSO-d6) δ 155.62, 147.94, 146.19, 145.76, 142.55, 142.34, 138.55, 137.13, 131.65, 130.14, 129.27, 128.75, 127.83, 126.13, 121.62, 115.40, 105.33, 73.38, 65.67, 64.27, 24.37, 23.66. HRMS: calcd for C_22_H_20_ClN_4_O_2_ *m/z* (M + H) 407.1269; found 407.1264. LC-MS M/Z (M + H) 407.0.

## AFTER EPUB

*[This article was published on 29 August 2024 with an error in Table 1. The table was corrected in the current version, posted on 9 September 2024.]*

## SUPPLEMENTAL MATERIAL

Supplemental figures mbio.00897-24-s0001.pdf Fig. S1 to S9.

Supplemental tables mbio.00897-24-s0002.docx Tables S1, S2, and S4 to S7; caption for Table S3.

Table S3 mbio.00897-24-s0003.xlsx GuaB key selectivity residues across Bacteria clades.

ASM does not own the copyrights to Supplemental Material that may be linked to, or accessed through, an article. The authors have granted ASM a non-exclusive, world-wide license to publish the Supplemental Material files. Please contact the corresponding author directly for reuse.

## References

[1] Nathan C, Cars O. 2014. Antibiotic resistance--problems, progress, and prospects. N Engl J Med 371:1761–1763. doi:10.1056/NEJMp1408040 25271470

[2] Davies SC, Fowler T, Watson J, Livermore DM, Walker D. 2013. Annual Report of the Chief Medical Officer: infection and the rise of antimicrobial resistance. Lancet 381:1606–1609. doi:10.1016/S0140-6736(13)60604-2 23489756

[3] Mulani MS, Kamble EE, Kumkar SN, Tawre MS, Pardesi KR. 2019. Emerging strategies to combat ESKAPE pathogens in the Era of antimicrobial resistance: a review. Front Microbiol 10:539. doi:10.3389/fmicb.2019.00539 30988669 PMC6452778

[4] Tacconelli E, Carrara E, Savoldi A, Harbarth S, Mendelson M, Monnet DL, Pulcini C, Kahlmeter G, Kluytmans J, Carmeli Y, Ouellette M, Outterson K, Patel J, Cavaleri M, Cox EM, Houchens CR, Grayson ML, Hansen P, Singh N, Theuretzbacher U, Magrini N, WHO Pathogens Priority List Working Group. 2018. Discovery, research, and development of new antibiotics: the WHO priority list of antibiotic-resistant bacteria and tuberculosis. Lancet Infect Dis 18:318–327. doi:10.1016/S1473-3099(17)30753-3 29276051

[5] Tommasi R, Brown DG, Walkup GK, Manchester JI, Miller AA. 2015. ESKAPEing the labyrinth of antibacterial discovery. Nat Rev Drug Discov 14:529–542. doi:10.1038/nrd4572 26139286

[6] Bentley R. 2000. Mycophenolic acid: a one hundred year odyssey from antibiotic to immunosuppressant. Chem Rev 100:3801–3826. doi:10.1021/cr990097b 11749328

[7] Florey HW, Jennings MA. 1946. Mycophenolic acid; an antibiotic from Penicillium brevicompactum Dlerckx. Lancet 1:46–49. doi:10.1016/s0140-6736(46)90242-5 21010114

[8] Franklin TJ, Cook JM. 1969. The inhibition of nucleic acid synthesis by mycophenolic acid. Biochem J 113:515–524. doi:10.1042/bj1130515 5807210 PMC1184694

[9] Hedstrom L, Liechti G, Goldberg JB, Gollapalli DR. 2011. The antibiotic potential of prokaryotic IMP dehydrogenase inhibitors. Curr Med Chem 18:1909–1918. doi:10.2174/092986711795590129 21517780 PMC5036587

[10] Shaffer CL, Zhang EW, Dudley AG, Dixon B, Guckes KR, Breland EJ, Floyd KA, Casella DP, Algood HMS, Clayton DB, Hadjifrangiskou M. 2017. Purine biosynthesis metabolically constrains intracellular survival of uropathogenic Escherichia coli. Infect Immun 85:e00471-16. doi:10.1128/IAI.00471-16 PMC5203662 27795353

[11] Subashchandrabose S, Smith SN, Spurbeck RR, Kole MM, Mobley HLT. 2013. Genome-wide detection of fitness genes in uropathogenic Escherichia coli during systemic infection. PLoS Pathog 9:e1003788. doi:10.1371/journal.ppat.1003788 24339777 PMC3855560

[12] Santiago AE, Mann BJ, Qin A, Cunningham AL, Cole LE, Grassel C, Vogel SN, Levine MM, Barry EM. 2015. Characterization of Francisella tularensis Schu S4 defined mutants as live-attenuated vaccine candidates. Pathog Dis 73:ftv036. doi:10.1093/femspd/ftv036 25986219 PMC4462183

[13] Santiago AE, Cole LE, Franco A, Vogel SN, Levine MM, Barry EM. 2009. Characterization of rationally attenuated Francisella tularensis vaccine strains that harbor deletions in the guaA and guaB genes. Vaccine (Auckl) 27:2426–2436. doi:10.1016/j.vaccine.2009.02.073 PMC2716139 19368784

[14] Sarkar S, Roberts LW, Phan M-D, Tan L, Lo AW, Peters KM, Paterson DL, Upton M, Ulett GC, Beatson SA, Totsika M, Schembri MA. 2016. Comprehensive analysis of type 1 fimbriae regulation in fimB-null strains from the multidrug resistant Escherichia coli ST131 clone. Mol Microbiol 101:1069–1087. doi:10.1111/mmi.13442 27309594

[15] Valentino MD, Foulston L, Sadaka A, Kos VN, Villet RA, Santa Maria J, Lazinski DW, Camilli A, Walker S, Hooper DC, Gilmore MS. 2014. Genes contributing to Staphylococcus aureus fitness in abscess- and infection-related ecologies. MBio 5:e01729-14. doi:10.1128/mBio.01729-14 25182329 PMC4173792

[16] Samant S, Lee H, Ghassemi M, Chen J, Cook JL, Mankin AS, Neyfakh AA. 2008. Nucleotide biosynthesis is critical for growth of bacteria in human blood. PLoS Pathog 4:e37. doi:10.1371/journal.ppat.0040037 18282099 PMC2242838

[17] Kobayashi K, Ehrlich SD, Albertini A, Amati G, Andersen KK, Arnaud M, Asai K, Ashikaga S, Aymerich S, Bessieres P, et al.. 2003. Essential Bacillus subtilis genes. Proc Natl Acad Sci U S A 100:4678–4683. doi:10.1073/pnas.0730515100 12682299 PMC153615

[18] McFarland WC, Stocker BA. 1987. Effect of different purine auxotrophic mutations on mouse-virulence of a Vi-positive strain of Salmonella dublin and of two strains of Salmonella typhimurium. Microb Pathog 3:129–141. doi:10.1016/0882-4010(87)90071-4 2849016

[19] Le Breton Y, Mistry P, Valdes KM, Quigley J, Kumar N, Tettelin H, McIver KS. 2013. Genome-wide identification of genes required for fitness of group A Streptococcus in human blood. Infect Immun 81:862–875. doi:10.1128/IAI.00837-12 23297387 PMC3584890

[20] Noriega FR, Losonsky G, Lauderbaugh C, Liao FM, Wang JY, Levine MM. 1996. Engineered deltaguaB-A deltavirG Shigella flexneri 2a strain CVD 1205: construction, safety, immunogenicity, and potential efficacy as a mucosal vaccine. Infect Immun 64:3055–3061. doi:10.1128/iai.64.8.3055-3061.1996 8757833 PMC174187

[21] Kofoed EM, Yan D, Katakam AK, Reichelt M, Lin B, Kim J, Park S, Date SV, Monk IR, Xu M, Austin CD, Maurer T, Tan M-W. 2016. De novo guanine biosynthesis but not the riboswitch-regulated purine salvage pathway is required for Staphylococcus aureus infection in vivo. J Bacteriol 198:2001–2015. doi:10.1128/JB.00051-16 27161118 PMC4936099

[22] Kotloff KL, Simon JK, Pasetti MF, Sztein MB, Wooden SL, Livio S, Nataro JP, Blackwelder WC, Barry EM, Picking W, Levine MM. 2007. Safety and immunogenicity of CVD 1208S, a live, oral ΔguaBA Δsen Δset Shigella flexneri 2a vaccine grown on animal-free media. Hum Vaccin 3:268–275. doi:10.4161/hv.4746 17938573

[23] Russo TA, Jodush ST, Brown JJ, Johnson JR. 1996. Identification of two previously unrecognized genes (guaA and argC) important for uropathogenesis. Mol Microbiol 22:217–229. doi:10.1046/j.1365-2958.1996.00096.x 8930907

[24] Park Y, Pacitto A, Bayliss T, Cleghorn LAT, Wang Z, Hartman T, Arora K, Ioerger TR, Sacchettini J, Rizzi M, et al.. 2017. Essential but not vulnerable: indazole sulfonamides targeting inosine monophosphate dehydrogenase as potential leads against Mycobacterium tuberculosis. ACS Infect Dis 3:18–33. doi:10.1021/acsinfecdis.6b00103 27704782 PMC5972394

[25] Juvale K, Purushothaman G, Singh V, Shaik A, Ravi S, Thiruvenkatam V, Kirubakaran S. 2019. Identification of selective inhibitors of Helicobacter pylori IMPDH as a targeted therapy for the infection. Sci Rep 9:190. doi:10.1038/s41598-018-37490-x 30655593 PMC6336804

[26] Chacko S, Boshoff HIM, Singh V, Ferraris DM, Gollapalli DR, Zhang M, Lawson AP, Pepi MJ, Joachimiak A, Rizzi M, Mizrahi V, Cuny GD, Hedstrom L. 2018. Expanding benzoxazole-based inosine 5’-monophosphate dehydrogenase (IMPDH) inhibitor structure-activity as potential antituberculosis agents. J Med Chem 61:4739–4756. doi:10.1021/acs.jmedchem.7b01839 29746130 PMC6166404

[27] Hedstrom L. 2009. IMP dehydrogenase: structure, mechanism, and inhibition. Chem Rev 109:2903–2928. doi:10.1021/cr900021w 19480389 PMC2737513

[28] Hedstrom L. 2017. The bare essentials of antibiotic target validation. ACS Infect Dis 3:2–4. doi:10.1021/acsinfecdis.6b00185 28081610 PMC5520969

[29] Gollapalli DR, Macpherson IS, Liechti G, Gorla SK, Goldberg JB, Hedstrom L. 2010. Structural determinants of inhibitor selectivity in prokaryotic IMP dehydrogenases. Chem Biol 17:1084–1091. doi:10.1016/j.chembiol.2010.07.014 21035731 PMC2991053

[30] Makowska-Grzyska M, Kim Y, Maltseva N, Osipiuk J, Gu M, Zhang M, Mandapati K, Gollapalli DR, Gorla SK, Hedstrom L, Joachimiak A. 2015. A novel cofactor-binding mode in bacterial IMP dehydrogenases explains inhibitor selectivity. J Biol Chem 290:5893–5911. doi:10.1074/jbc.M114.619767 25572472 PMC4342496

[31] Makowska-Grzyska M, Kim Y, Gorla SK, Wei Y, Mandapati K, Zhang M, Maltseva N, Modi G, Boshoff HI, Gu M, Aldrich C, Cuny GD, Hedstrom L, Joachimiak A. 2015. Mycobacterium tuberculosis IMPDH in complexes with substrates, products and antitubercular compounds. PLoS ONE 10:e0138976. doi:10.1371/journal.pone.0138976 26440283 PMC4594927

[32] Hug LA, Baker BJ, Anantharaman K, Brown CT, Probst AJ, Castelle CJ, Butterfield CN, Hernsdorf AW, Amano Y, Ise K, Suzuki Y, Dudek N, Relman DA, Finstad KM, Amundson R, Thomas BC, Banfield JF. 2016. A new view of the tree of life. Nat Microbiol 1:16048. doi:10.1038/nmicrobiol.2016.48 27572647

[33] Williams KP, Gillespie JJ, Sobral BWS, Nordberg EK, Snyder EE, Shallom JM, Dickerman AW. 2010. Phylogeny of gammaproteobacteria. J Bacteriol 192:2305–2314. doi:10.1128/JB.01480-09 20207755 PMC2863478

[34] Morrow CA, Valkov E, Stamp A, Chow EWL, Lee IR, Wronski A, Williams SJ, Hill JM, Djordjevic JT, Kappler U, Kobe B, Fraser JA. 2012. De novo GTP biosynthesis is critical for virulence of the fungal pathogen Cryptococcus neoformans. PLoS Pathog 8:e1002957. doi:10.1371/journal.ppat.1002957 23071437 PMC3469657

[35] Singh V, Donini S, Pacitto A, Sala C, Hartkoorn RC, Dhar N, Keri G, Ascher DB, Mondésert G, Vocat A, Lupien A, Sommer R, Vermet H, Lagrange S, Buechler J, Warner DF, McKinney JD, Pato J, Cole ST, Blundell TL, Rizzi M, Mizrahi V. 2017. The inosine monophosphate dehydrogenase, GuaB2, is a vulnerable new bactericidal drug target for tuberculosis. ACS Infect Dis 3:5–17. doi:10.1021/acsinfecdis.6b00102 27726334 PMC5241705

[36] Cox JAG, Mugumbate G, Del Peral L-G, Jankute M, Abrahams KA, Jervis P, Jackenkroll S, Perez A, Alemparte C, Esquivias J, Lelièvre J, Ramon F, Barros D, Ballell L, Besra GS. 2016. Novel inhibitors of Mycobacterium tuberculosis GuaB2 identified by a target based high-throughput phenotypic screen. Sci Rep 6:38986. doi:10.1038/srep38986 27982051 PMC5159837

[37] Trapero A, Pacitto A, Singh V, Sabbah M, Coyne AG, Mizrahi V, Blundell TL, Ascher DB, Abell C. 2018. Fragment-based approach to targeting inosine-5’-monophosphate dehydrogenase (IMPDH) from Mycobacterium tuberculosis. J Med Chem 61:2806–2822. doi:10.1021/acs.jmedchem.7b01622 29547284 PMC5900554

[38] Rao VA, Shepherd SM, Owen R, Hunter WN. 2013. Structure of Pseudomonas aeruginosa inosine 5’-monophosphate dehydrogenase. Acta Cryst Sect F Struct Biol Cryst Commun 69:243–247. doi:10.1107/S1744309113002352 PMC3606566 23519796

[39] Zgurskaya HI, Löpez CA, Gnanakaran S. 2015. Permeability barrier of Gram-negative cell envelopes and approaches to bypass it. ACS Infect Dis 1:512–522. doi:10.1021/acsinfecdis.5b00097 26925460 PMC4764994

[40] Bohnert T, Gan LS. 2013. Plasma protein binding: from discovery to development. J Pharm Sci 102:2953–2994. doi:10.1002/jps.23614 23798314

[41] Silver LL. 2011. Challenges of antibacterial discovery. Clin Microbiol Rev 24:71–109. doi:10.1128/CMR.00030-10 21233508 PMC3021209

[42] O’Dwyer K, Spivak AT, Ingraham K, Min S, Holmes DJ, Jakielaszek C, Rittenhouse S, Kwan AL, Livi GP, Sathe G, Thomas E, Van Horn S, Miller LA, Twynholm M, Tomayko J, Dalessandro M, Caltabiano M, Scangarella-Oman NE, Brown JR. 2015. Bacterial resistance to leucyl-tRNA synthetase inhibitor GSK2251052 develops during treatment of complicated urinary tract infections. Antimicrob Agents Chemother 59:289–298. doi:10.1128/AAC.03774-14 25348524 PMC4291364

[43] Luria SE, Delbrück M. 1943. Mutations of bacteria from virus sensitivity to virus resistance. Genetics 28:491–511. doi:10.1093/genetics/28.6.491 17247100 PMC1209226

[44] Foster PL. 2006. Methods for determining spontaneous mutation rates. Methods Enzymol 409:195–213. doi:10.1016/S0076-6879(05)09012-9 16793403 PMC2041832

[45] Tucker AT, Nowicki EM, Boll JM, Knauf GA, Burdis NC, Trent MS, Davies BW. 2014. Defining gene-phenotype relationships in Acinetobacter baumannii through one-step chromosomal gene inactivation. MBio 5:e01313-14. doi:10.1128/mBio.01313-14 25096877 PMC4128354

[46] Smart OS, Womack TO, Flensburg C, Keller P, Paciorek W, Sharff A, Vonrhein C, Bricogne G. 2011. Better ligand representation in BUSTER protein-complex structure determination. Acta Crystallogr A Found Crystallogr 67:C134–C134. doi:10.1107/S010876731109670X

[47] Camacho C, Coulouris G, Avagyan V, Ma N, Papadopoulos J, Bealer K, Madden TL. 2009. BLAST+: architecture and applications. BMC Bioinformatics 10:421. doi:10.1186/1471-2105-10-421 20003500 PMC2803857

[48] Katoh K, Standley DM. 2013. MAFFT multiple sequence alignment software version 7: improvements in performance and usability. Mol Biol Evol 30:772–780. doi:10.1093/molbev/mst010 23329690 PMC3603318

[49] Emsley P, Cowtan K. 2004. Coot: model-building tools for molecular graphics. Acta Cryst Sect D: Biol Cryst 60:2126–2132. 10.1107/S0907444904019158 15572765

[50] Adams PD, Afonine PV, Bunkóczi G, Chen VB, Davis IW, Echols N, Headd JJ, Hung L-W, Kapral GJ, Grosse-Kunstleve RW, McCoy AJ, Moriarty NW, Oeffner R, Read RJ, Richardson DC, Richardson JS, Terwilliger TC, Zwart PH. 2010. PHENIX: a comprehensive Python-based system for macromolecular structure solution. Acta Crystallogr D Biol Crystallogr 66:213–221. doi:10.1107/S0907444909052925 20124702 PMC2815670

[51] Sastry GM, Adzhigirey M, Day T, Annabhimoju R, Sherman W. 2013. Protein and ligand preparation: parameters, protocols, and influence on virtual screening enrichments. J Comput Aided Mol Des 27:221–234. doi:10.1007/s10822-013-9644-8 23579614

[52] Lu C, Wu C, Ghoreishi D, Chen W, Wang L, Damm W, Ross GA, Dahlgren MK, Russell E, Von Bargen CD, Abel R, Friesner RA, Harder ED. 2021. OPLS4: improving force field accuracy on challenging regimes of chemical space. J Chem Theory Comput 17:4291–4300. doi:10.1021/acs.jctc.1c00302 34096718

[53] Abel R, Young T, Farid R, Berne BJ, Friesner RA. 2008. Role of the active-site solvent in the thermodynamics of factor Xa ligand binding. J Am Chem Soc 130:2817–2831. doi:10.1021/ja0771033 18266362 PMC2761766

[54] Young T, Abel R, Kim B, Berne BJ, Friesner RA. 2007. Motifs for molecular recognition exploiting hydrophobic enclosure in protein–ligand binding. Proc Natl Acad Sci U S A 104:808–813. doi:10.1073/pnas.0610202104 17204562 PMC1783395

