# Supplementary material for: Discovery of GuaB inhibitors with efficacy against Acinetobacter baumannii infection
Source: mBio. 2024 Aug 29;15(10):e00897-24. doi: 10.1128/mbio.00897-24 (PMC11481871; doi:10.1128/mbio.00897-24)
Supplement: Supplemental figures — Fig. S1 to S9. [file mbio.00897-24-s0001.pdf]

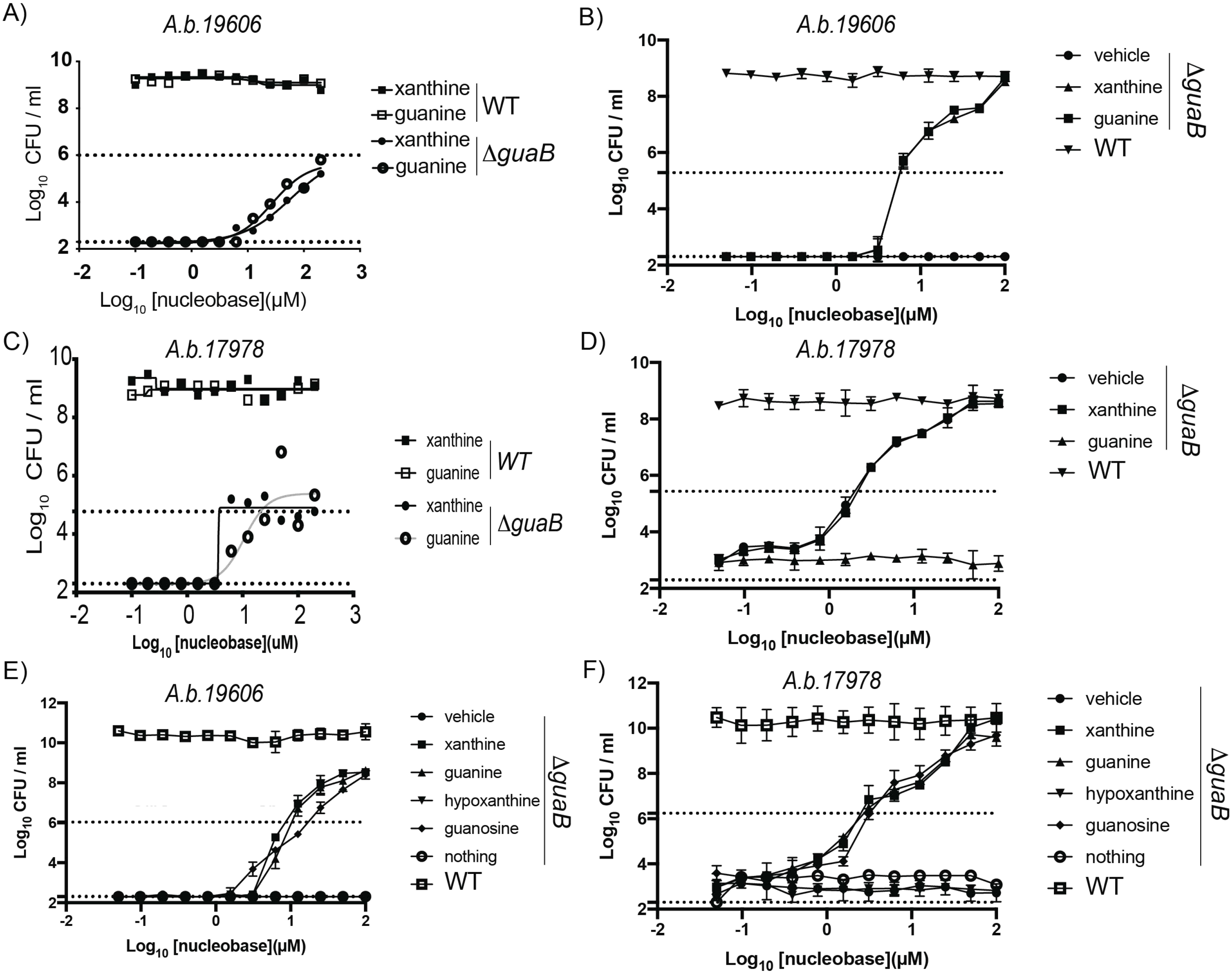

Supplementary Figure S1: Exogenous nucleobases can be salvaged and utilized to rescue the loss of viability of A. baumannii ΔguaB mutants in vitro. (A) A.b.19606 wild-type and ΔguaB were grown overnight at 37C on tryptic soy agar (TSA), resuspended into liquid cultures containing a dose response of (0-200 μM) of xanthine or guanine, and colony forming units (CFUs) were enumerated by plating on TSA 24h later. (B) A.b.19606 wild-type and ΔguaB were grown overnight at 37C on TSA + 1mM guanine, resuspended into liquid cultures containing dose response (0-100 μM) of vehicle, xanthine, or guanine and CFUs were enumerated by plating on TSA+1mM guanine 24h later. (C) A.b.17978 wild-type and ΔguaB were grown overnight at 37C on TSA, resuspended into liquid cultures containing dose response of xanthine and guanine and viability was determined as in (A) above. (D) A.b.17978 wild-type and ΔguaB were grown at 37C overnight on TSA+1mM guanine, resuspended into liquid cultures containing dose response of vehicle, xanthine, and guanine and viability was determined as in (B) above. (E) A.b.19606 wild-type and ΔguaB were grown at 37C overnight on TSA+1mM guanine, resuspended into liquid cultures containing a dose response (0-100μM) of xanthine, guanine, hypoxanthine, guanosine, or controls and CFUs were enumerated the next day on TSA+1mM guanine. (F) A.b.17978 wild-type and ΔguaB were grown at 37C overnight on TSA+1mM guanine, resuspended in liquid culture containing a dose response (0-100μM) of xanthine, guanine, hypoxanthine, guanosine, or controls and CFUs were enumerated the next day on TSA+1mM guanine. Dotted lines indicate bacterial input, and assay lower limit of quantification (LLOQ). Nucleobases were prepared at 100mM stocks in 0.2N NaOH, and diluted directly into media.

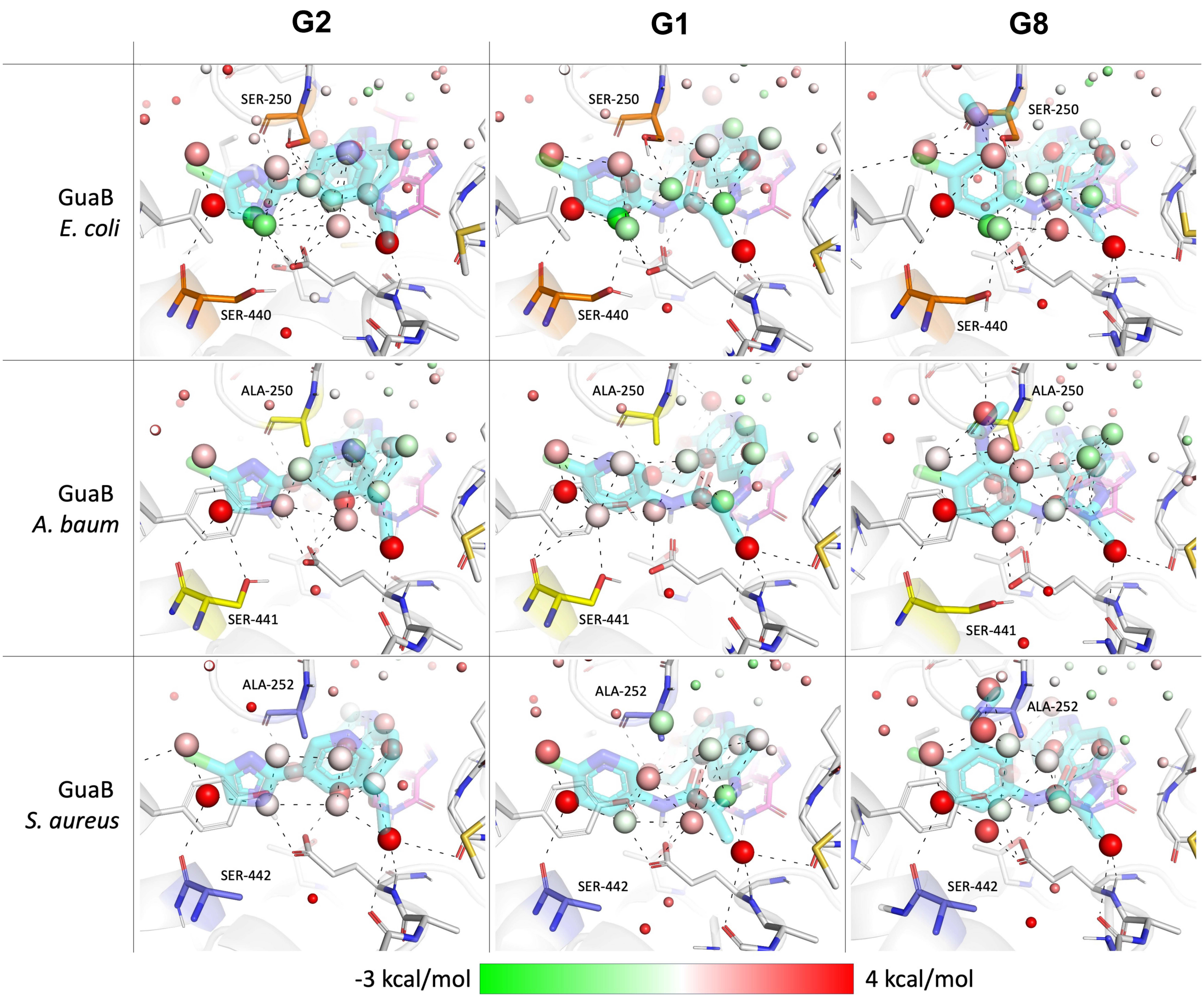

Supplementary Figure S2. WaterMap analysis for the protein water network in the binding site of GuaB *E. coli* (upper panel), GuaB *A. baumannii* (middle panel) and GuaB *S. aureus* (lower panel). The key residues: ALA-442 and ALA-252 in GuaB *S. aureus*, SER-441 and ALA-250 in GuaB *A. baumannii*, and SER-440 and SER-250 in GuaB *E. coli* are colored pale blue, yellow and orange respectively. Predicted hydration sites are shown as spheres and colored by their desolvation free energy (-3 kcal/mol to 4 kcal/mol) in the binding site. The larger spheres are within 1Å to the bound ligand (transparent sticks in cyan) highlighting the water molecules that will be displaced upon ligand binding. Black dashes indicate the hypothetical water network that will be disrupted.

Supplementary Figure S3

A)

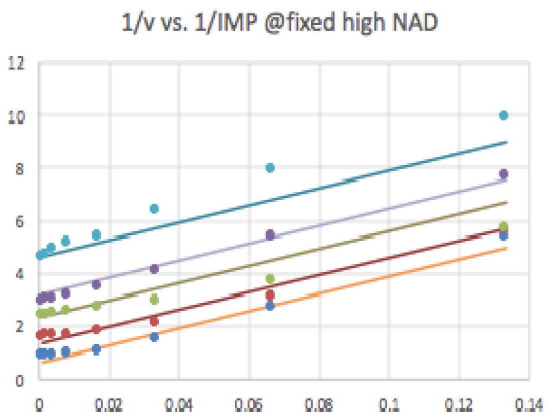

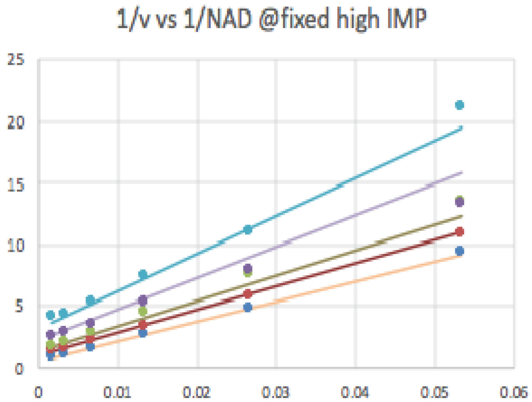

G2 stereoisomer

Uncompetitive vs IMP
K3*=23 nM

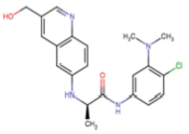

Mixed Type vs NAD
K2=298 nM
K3*=29 nM

* The two K3 in theory should be identical. Here the two numbers are close enough to be the same.

B)

GuaB: IMP Dehydrogenase

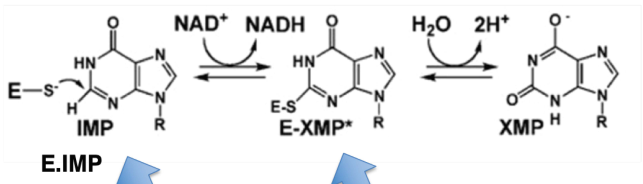

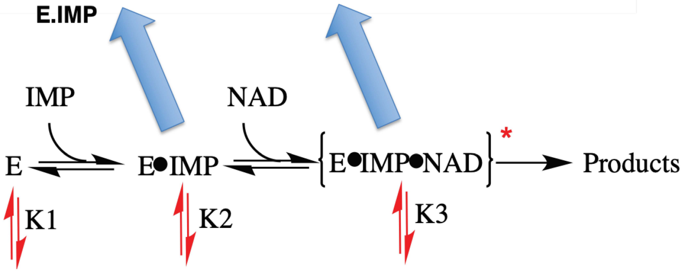

- *Kinetically, the {E.IMP.NAD } complex term contains all enzyme forms after the E.IMP binary complex, including E-XMP.NADH, E-XMP intermediate, E.XMP, etc.
- In GuaB case, K1 is infinitely high meaning no binding of to the apo form of the enzyme

Supplementary Figure S3: (A) The mechanism of inhibition of G2 against *A. baumannii* GuaB was determined using the less potent stereoisomer of G2 by generating Lineweaver Burk plots. The mechanism of inhibition was uncompetitive versus substrate IMP, and displayed a mixed non-competitive binding with NAD+. (B) GuaBi can bind both the Enzyme-IMP, and Enzyme-XMP complexes. Kinetically, the E.IMP.NAD complex term contains all enzyme forms after the E.IMP binary complex, including E-XMP.NADH, E-XMP intermediate, E.XMP, etcetera. In the case of GuaB, K1 is infinitely high meaning no binding of the apo form of the enzyme. Depiction of catalytic mechanism was derived from Hedstrom et al., 2009; (Chemical Reviews).

Supplementary Figure S4

A) **G2 Pharmacokinetic Profile in Mice**

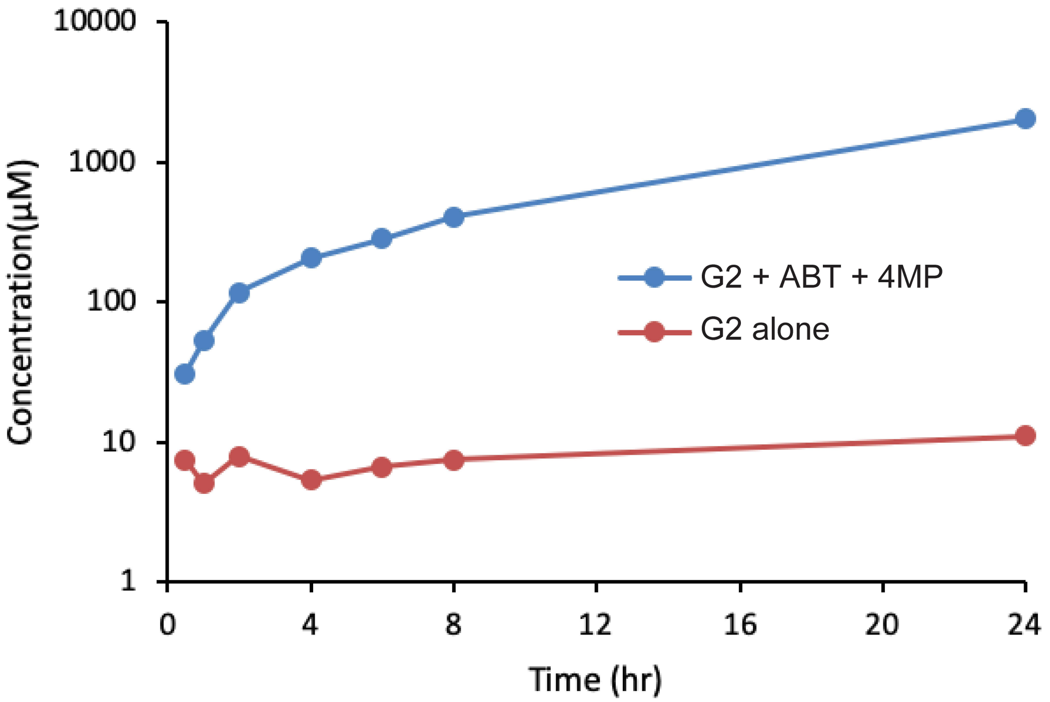

B) **G1 Pharmacokinetic Profile in Mice**

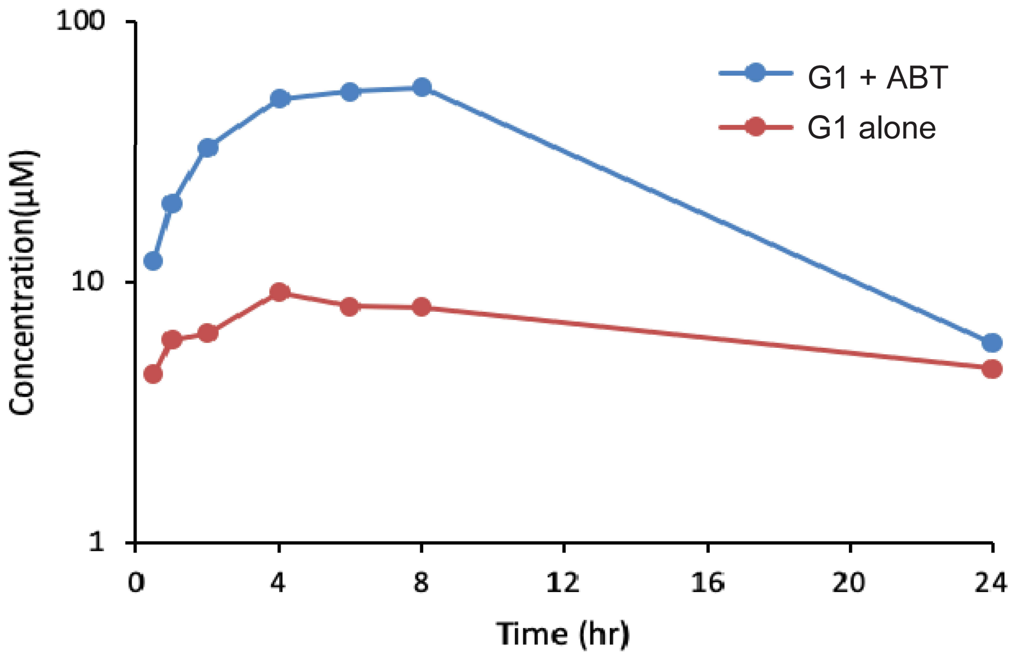

Supplementary Figure S4: Pharmacokinetics of G2 and G1 in mice. (A) Pharmacokinetics of intravenous delivered G2 alone or co-administered with 1-aminobenzotriazole (ABT) and 4-methylpyrazole over 24 hours. (B) Pharmacokinetics of intravenous delivered G1 alone or co-administered with ABT. Each data point represents the blood concentration of GuaB inhibitor at the indicated time.

Supplementary Figure S5

A)

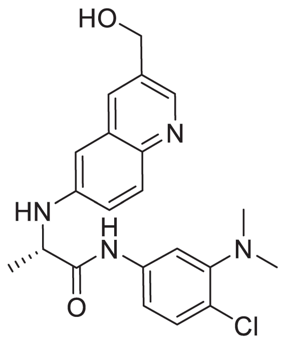

G2
mPPB = 88%
MIC = 2.4uM

B) GuaBi G2 Bactericidal Efficacy against *Acinetobacter baumannii* 19606 in neutropenic thigh infection model

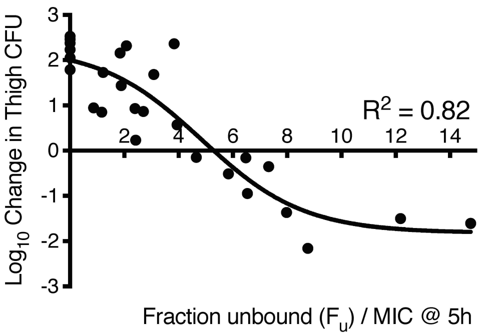

C)

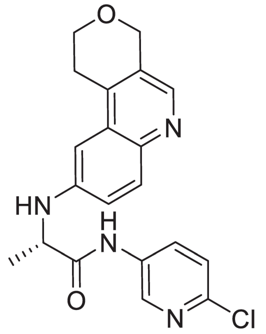

G1
mPPB = 95%
MIC = 0.7uM

D) GuaBi G1 Bactericidal Efficacy against *Acinetobacter baumannii* 19606 in neutropenic thigh infection model

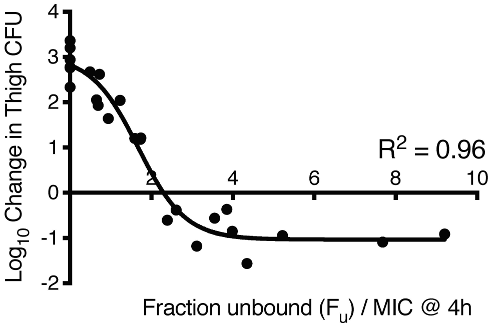

Supplementary Figure S5: Pharmakodynamic activity of G2 and G1 against *Acinetobacter baumannii* 19606 mouse neutropenic thigh infection. (A) Structure, measured plasma protein binding (mPPB), and serum-free MIC of G2. (B) Plot of the change in thigh bacterial load as a function of the fraction unbound of G2 in blood measured by LCMS at 5 hours over the MIC. (C) Structure, mPPB, and serum-free MIC of G1. (D) Plot of the change in thigh bacterial load as a function of the fraction unbound of G1 in blood at 4 hours over the MIC. Data in (B) and (D) are from two independent experiments and multiple dosing groups combined. Each data point represents a single mouse, and change in CFU was the difference between bacterial load at pre-treatment and 22 hours later.

Supplementary Figure S6

A) G2_*A. baumannii* MICs in M9 (38 isolates; Avg. of n=3 experiments)

B) G1_*A. baumannii* MICs in M9 (38 isolates; Avg. of n=3 experiments)

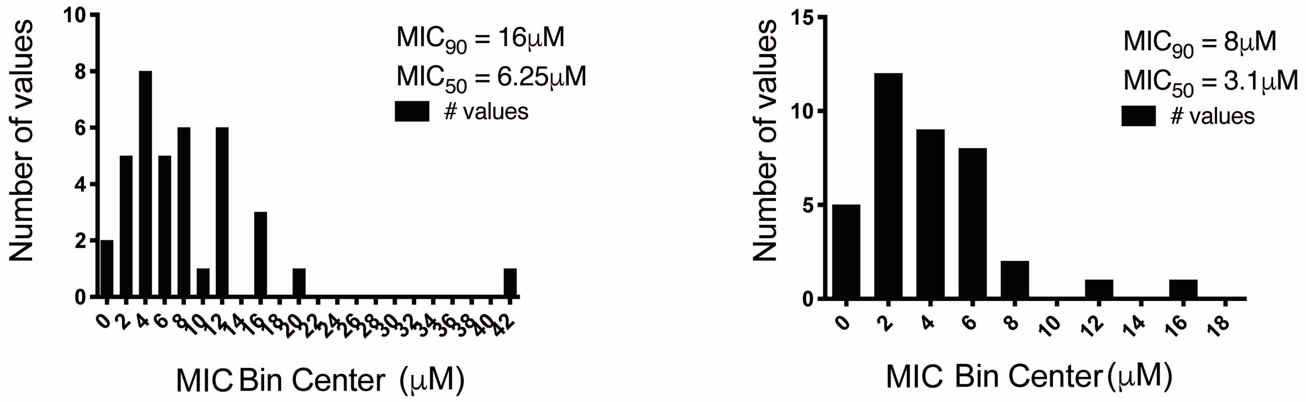

Supplementary Figure S6: Frequency distributions of minimal inhibitory concentration (MIC) of (A) G2 and (B) G1 across 38 different multi-drug resistant (MDR) *A. baumannii* clinical isolates obtained from the Centers for Disease Control and Prevention (CDC) and International Health Management Associates (IHMA) carbapenem-resistant isolate panels. MIC values were determined using the Clinical and Laboratory Standards Institute (CLSI) microdilution protocol with modification using M9 media, incubated at 37C for 20-24h and read visually. The x-axes represent MIC values in microMolar, and the y-axes indicate the number of isolates that have that MIC value for compound G1, or G2. The MIC_{50} and MIC_{90} values refer to the lowest concentration of inhibitor that prevents growth of bacteria in 50% and 90% of isolates, respectively.

G1

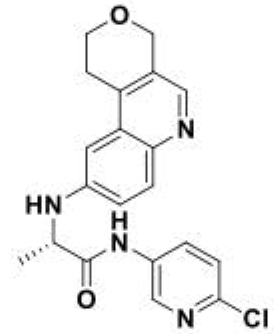

(2S)-N-(6-Chloro-3-pyridyl)-2-(2,4-dihydro-1H-pyrano[3,4-c]quinolin-9 ylamino)propanamide

Scheme:

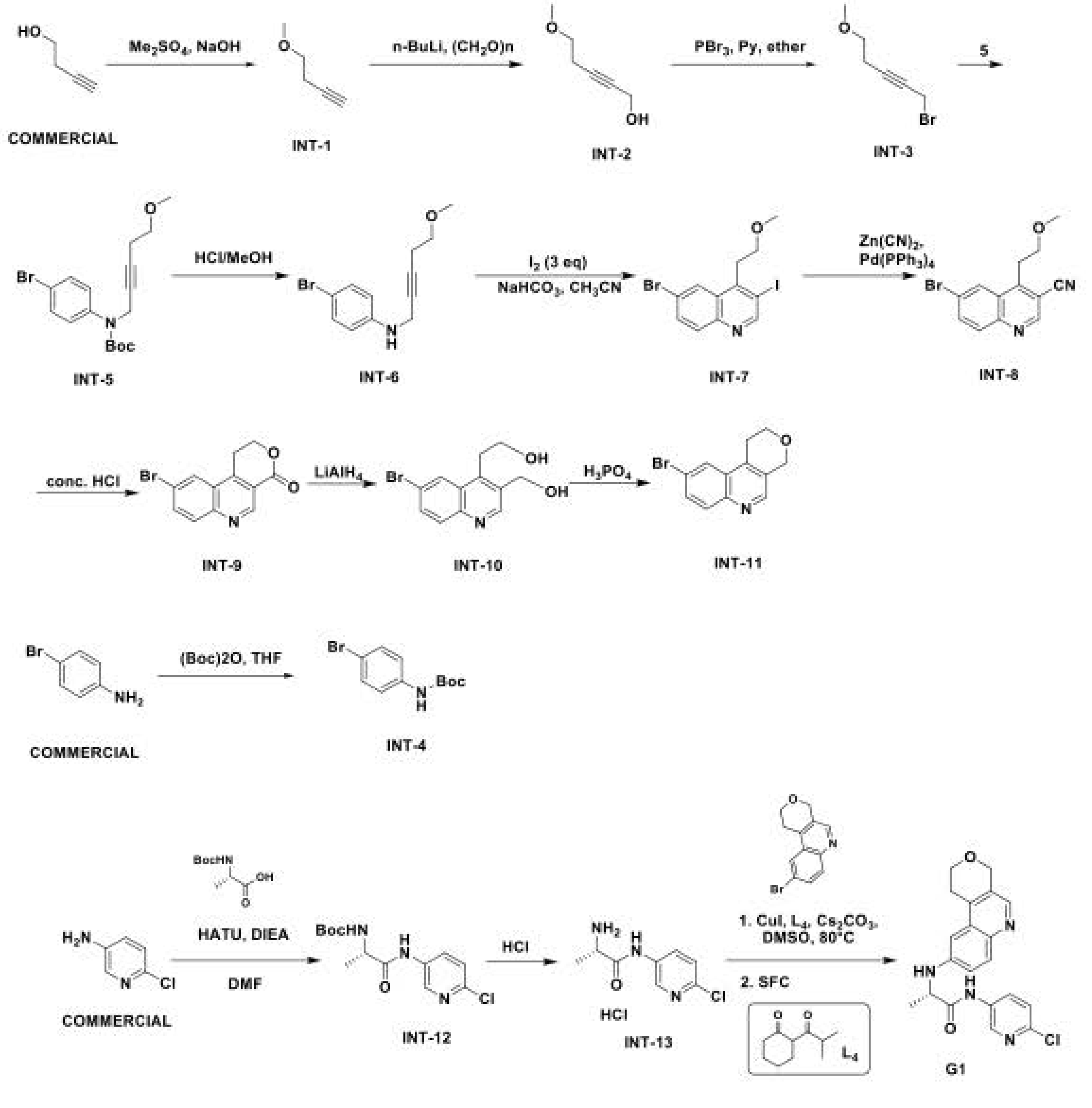

Supplementary Figure S7: Synthesis of G1. See methods for details.

G2

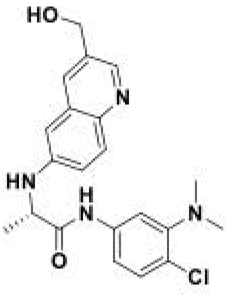

(2S)-N-[4-Chloro-3-(dimethylamino)phenyl]-2-[[3-(hydroxymethyl)-6-quinolyl]amino]propanamide

Scheme:

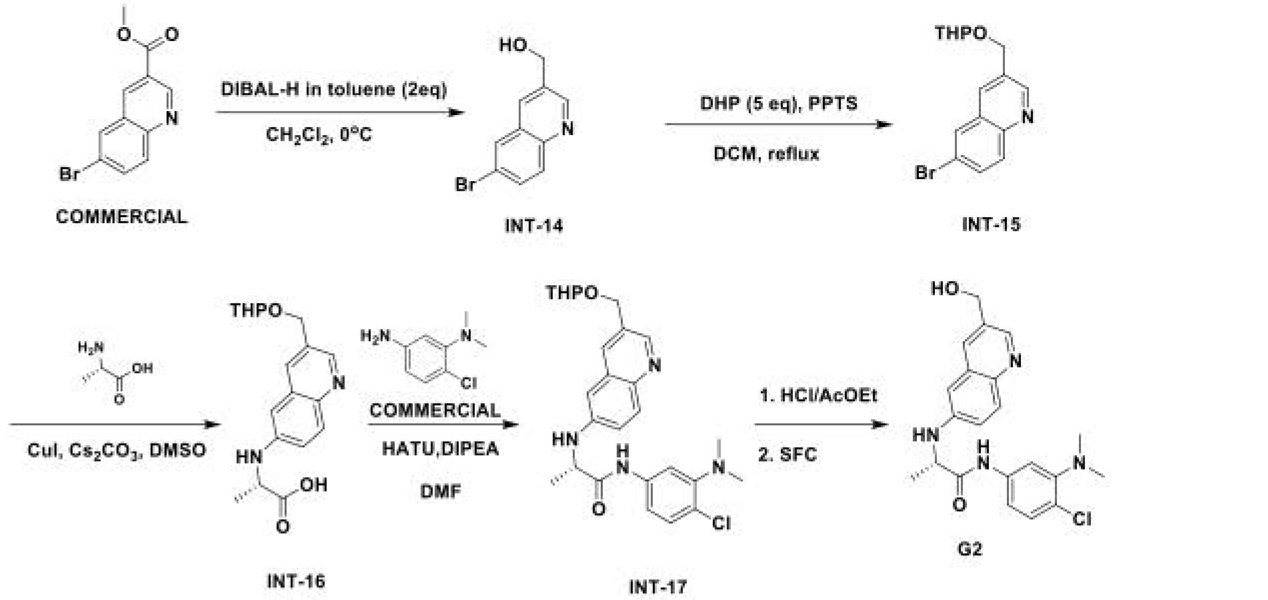

Supplementary Figure S8: Synthesis of G2. See methods section for chemistry experimental details.

G8

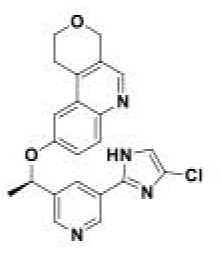

(R)-9-[1-[5-(4-Chloro-1H-imidazol-2-yl)-3-pyridyl]ethoxy]-2,4-dihydro-1H-pyrano[3,4-c]quinoline

Scheme:

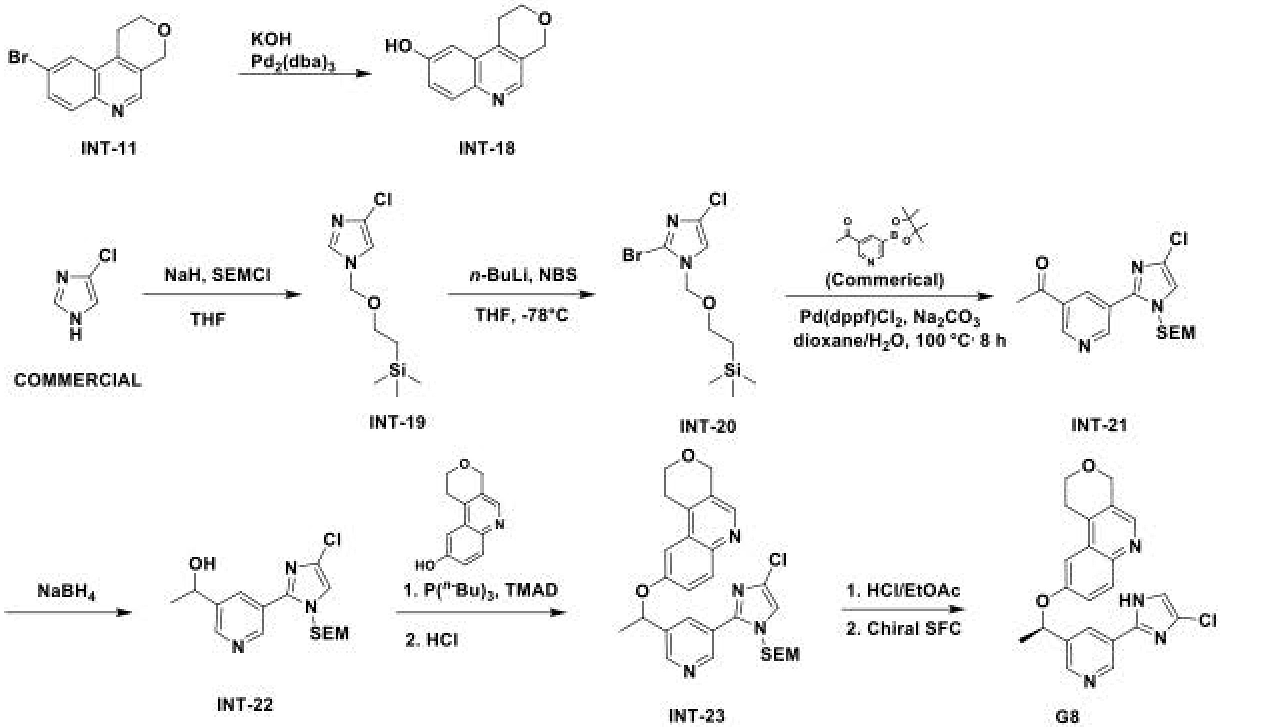

Supplementary Figure S9: Synthesis of G8. See methods section for chemistry experimental details.
