# Supplementary material for: Discovery of GuaB inhibitors with efficacy against Acinetobacter baumannii infection
Source: mBio. 2024 Aug 29;15(10):e00897-24. doi: 10.1128/mbio.00897-24 (PMC11481871; doi:10.1128/mbio.00897-24)
Supplement: Supplemental tables — Tables S1, S2, and S4 to S7; caption for Table S3. [file mbio.00897-24-s0002.docx]

**Supplementary Table S1**. Purine concentrations in mouse and human serum determined by LC-MS assay. Calibration curve ranges: guanosine (0.034-17.65μM), guanine (1.036-33.091μM), xanthine (0.064-18.641μM), inosine (0.009-36.735μM), hypoxanthine (0.036-36.735μM); BLOQ indicates samples were below the level of quantification.

| **Average Plasma Concentration (µM) +/- S.D.** | **Guanosine** | **Guanine** | **Xanthine** | **Inosine** | **Hypoxanthine** |
| --- | --- | --- | --- | --- | --- |
| Human (n=3) | BLOQ | BLOQ | 0.364±0.100 | 0.015±0.001 | 1.278±0.522 |
| Mouse CD-1 (n=6) | BLOQ | BLOQ | 0.704±0.556 | 0.772±0.527 | 0.358±0.255 |
| Mouse A/J (n=5) | BLOQ | BLOQ | 1.054±0.343 | 0.554±0.602 | 0.388±0.274 |

**Supplementary Table S2.** Data Collection and refinement statistics for (A) GuaB:G1 complexes in 3 species, (B) GuaB:G2 complexes in 3 species, and (C) GuaB:G8 complexes in 3 species.

**Supplementary Table S2A:** Data collection and refinement statistics for GuaB:G1 complexes in 3 species.

| **Species GuaB : G1** | **AbG1 (9AUV)** | **SaG1 (9AUY)** | **EcG1 (9AV1)** |
| --- | --- | --- | --- |
| **Wavelength** | 0.9795 | 0.9792 | 0.9792 |
| **Resolution range** | 46.54 - 1.83 (1.895 -1.83) | 52.14 - 1.94 (2.009 -1.94) | 80.25 - 1.7 (1.761 -1.7) |
| **Space group** | P 1 21 1 | I 4 | I 4 |
| **Unit cell** | 100.731 127.277 126.702 90 104.781 90 | 104.279 104.279 63.903 90 90 90 | 113.485 113.485 55.475 90 90 90 |
| **Total reflections** | 849696 (84582) | 168270 (16659) | 259681 (25997) |
| **Unique reflections** | 247998 (25646) | 25134 (2486) | 38864 (3846) |
| **Multiplicity** | 3.4 (3.3) | 6.7 (6.7) | 6.7 (6.8) |
| **Completeness (%)** | 91.40 (94.77) | 98.52 (98.03) | 99.34 (99.95) |
| **Mean I/sigma(I)** | 13.46 (2.40) | 26.14 (1.96) | 24.19 (1.84) |
| **Wilson B-factor** | 23.37 | 49.37 | 31.20 |
| **R-merge** | 0.05837 (0.5148) | 0.03106 (1.007) | 0.03928 (0.9162) |
| **R-meas** | 0.06936 (0.618) | 0.03372 (1.093) | 0.04263 (0.9919) |
| **R-pim** | 0.03693 (0.3358) | 0.01297 (0.4201) | 0.01639 (0.3773) |
| **CC1/2** | 0.991 (0.818) | 1 (0.706) | 1 (0.725) |
| **CC*** | 0.998 (0.949) | 1 (0.91) | 1 (0.917) |
| **Reflections used in refinement** | 247948 (25637) | 25108 (2486) | 38689 (3846) |
| **Reflections used for R-free** | 12527 (1275) | 1248 (118) | 1949 (213) |
| **R-work** | 0.1348 (0.2334) | 0.2324 (0.3455) | 0.2190 (0.3684) |
| **R-free** | 0.1865 (0.2935) | 0.2722 (0.4061) | 0.2592 (0.4007) |
| **CC(work)** | 0.977 (0.935) | 0.937 (0.655) | 0.956 (0.680) |
| **CC(free)** | 0.959 (0.865) | 0.958 (0.548) | 0.943 (0.616) |
| **Number of non-hydrogen atoms** | 22826 | 2671 | 2640 |
| macromolecules | 20469 | 2562 | 2383 |
| ligands | 216 | 27 | 27 |
| solvent | 2141 | 82 | 230 |
| Protein residues | 2747 | 326 | 326 |
| RMS(bonds) | 0.004 | 0.014 | 0.014 |
| RMS(angles) | 0.64 | 1.72 | 1.70 |
| Ramachandran favored (%) | 97.56 | 98.14 | 95.96 |
| Ramachandran allowed (%) | 2.44 | 1.86 | 3.73 |
| Ramachandran outliers (%) | 0.00 | 0.00 | 0.31 |
| Rotamer outliers (%) | 1.95 | 4.15 | 6.67 |
| Clashscore | 6.72 | 1.92 | 3.51 |
| Average B-factor | 33.45 | 61.52 | 39.45 |
| macromolecules | 31.87 | 61.78 | 38.80 |
| ligands | 24.68 | 51.01 | 33.71 |
| solvent | 49.49 | 57.06 | 46.88 |

Statistics for the highest-resolution shell are shown in parentheses.

**Supplementary Table S2B.** Data collection and refinement statistics for GuaB:G2 complexes in 3 species.

| **Species GuaB : G2** | | **AbG2 (9AUW)** | | **SaG2 (9AUZ)** | **EcG2 (9AV2)** |
| --- | --- | --- | --- | --- | --- |
| **Wavelength** | 0.9792 | | 0.9792 | | 0.9792 |
| **Resolution range** | 44.55 - 2.3 (2.382 -2.3) | | 54.54 - 1.94 (2.01 -1.94) | | 22.66 - 1.7 (1.761 -1.7) |
| **Space group** | P 1 21 1 | | I 4 | | I 4 |
| **Unit cell** | 113.72 127.299 127.478 90 101.825 90 | | 100.627 100.627 64.907 90 90 90 | | 113.343 113.343 54.964 90 90 90 |
| **Total reflections** | 953755 (52483) | | 149353 (13711) | | 241464 (25784) |
| **Unique reflections** | 156123 (15338) | | 23906 (2195) | | 37434 (3829) |
| **Multiplicity** | 6.1 (3.4) | | 6.2 (5.7) | | 6.5 (6.7) |
| **Completeness (%)** | 99.01 (97.73) | | 98.24 (90.85) | | 97.10 (99.92) |
| **Mean I/sigma(I)** | 2.66 (1.09) | | 8.99 (1.97) | | 12.94 (1.79) |
| **Wilson B-factor** | 24.30 | | 29.33 | | 30.18 |
| **R-merge** | 0.3758 (0.6087) | | 0.1149 (0.9545) | | 0.07269 (0.8705) |
| **R-meas** | 0.4103 (0.7233) | | 0.1253 (1.049) | | 0.07927 (0.9438) |
| **R-pim** | 0.1624 (0.3859) | | 0.04914 (0.4288) | | 0.03118 (0.3621) |
| **CC1/2** | 0.935 (0.799) | | 0.996 (0.875) | | 0.997 (0.777) |
| **CC*** | 0.983 (0.943) | | 0.999 (0.966) | | 0.999 (0.935) |
| **Reflections used in refinement** | 156053 (15338) | | 23682 (2194) | | 37415 (3829) |
| **Reflections used for R-free** | 7966 (776) | | 1170 (79) | | 1883 (203) |
| **R-work** | 0.2929 (0.3437) | | 0.2192 (0.3558) | | 0.2113 (0.4084) |
| **R-free** | 0.3147 (0.3758) | | 0.2566 (0.3896) | | 0.2594 (0.4249) |
| **CC(work)** | 0.836 (0.641) | | 0.931 (0.888) | | 0.959 (0.772) |
| **CC(free)** | 0.818 (0.560) | | 0.957 (0.801) | | 0.934 (0.710) |
| **Number of non-hydrogen atoms** | 20630 | | 2647 | | 2657 |
| macromolecules | 19961 | | 2548 | | 2419 |
| ligands | 224 | | 28 | | 28 |
| solvent | 445 | | 71 | | 210 |
| Protein residues | 2692 | | 326 | | 328 |
| RMS(bonds) | 0.013 | | 0.014 | | 0.014 |
| RMS(angles) | 1.69 | | 1.68 | | 1.69 |
| Ramachandran favored (%) | 97.69 | | 97.52 | | 97.22 |
| Ramachandran allowed (%) | 2.27 | | 2.48 | | 2.78 |
| Ramachandran outliers (%) | 0.04 | | 0.00 | | 0.00 |
| Rotamer outliers (%) | 4.22 | | 4.18 | | 3.69 |
| Clashscore | 2.74 | | 1.35 | | 2.64 |
| Average B-factor | 28.18 | | 38.93 | | 40.60 |
| macromolecules | 28.32 | | 38.94 | | 40.12 |
| ligands | 24.64 | | 40.56 | | 35.21 |
| solvent | 23.91 | | 38.11 | | 46.78 |

Statistics for the highest-resolution shell are shown in parentheses.

**Supplementary Table S2C.** Data collection and refinement statistics for GuaB:G8 complexes in 3 species.

| **Species GuaB : G8** | | **AbG8 (9AUX)** | | **SaG8 (9AV0)** | **EcG8 (9AV3)** |
| --- | --- | --- | --- | --- | --- |
| **Wavelength** | 0.9791 | | 0.9792 | | 0.9792 |
| **Resolution range** | 91.92 - 2.46 (2.548 -2.46) | | 52.22 - 2.2 (2.279 -2.2) | | 49.88 - 1.82 (1.885 -1.82) |
| **Space group** | P 1 21 1 | | I 4 | | I 4 |
| **Unit cell** | 112.689 126.412 126.387 90 101.32 90 | | 104.442 104.442 64.413 90 90 90 | | 113.76 113.76 55.502 90 90 90 |
| **Total reflections** | 422252 (43658) | | 114952 (10103) | | 210648 (21372) |
| **Unique reflections** | 124097 (12188) | | 17437 (1518) | | 31098 (3093) |
| **Multiplicity** | 3.4 (3.5) | | 6.6 (6.7) | | 6.8 (6.9) |
| **Completeness (%)** | 98.06 (97.33) | | 98.34 (86.30) | | 97.24 (97.85) |
| **Mean I/sigma(I)** | 7.68 (2.90) | | 18.38 (2.00) | | 20.07 (1.85) |
| **Wilson B-factor** | 27.69 | | 60.42 | | 33.22 |
| **R-merge** | 0.1517 (0.4218) | | 0.05406 (0.9369) | | 0.05317 (0.992) |
| **R-meas** | 0.1811 (0.5002) | | 0.05889 (1.016) | | 0.05761 (1.071) |
| **R-pim** | 0.09752 (0.2657) | | 0.02308 (0.3892) | | 0.02196 (0.4002) |
| **CC1/2** | 0.981 (0.879) | | 0.999 (0.658) | | 0.999 (0.77) |
| **CC*** | 0.995 (0.967) | | 1 (0.891) | | 1 (0.933) |
| **Reflections used in refinement** | 123545 (12186) | | 17435 (1518) | | 31095 (3092) |
| **Reflections used for R-free** | 6317 (676) | | 905 (86) | | 1544 (141) |
| **R-work** | 0.1968 (0.2585) | | 0.2484 (0.3751) | | 0.2062 (0.3358) |
| **R-free** | 0.2497 (0.3343) | | 0.2865 (0.4083) | | 0.2466 (0.3581) |
| **CC(work)** | 0.941 (0.908) | | 0.919 (0.457) | | 0.959 (0.710) |
| **CC(free)** | 0.904 (0.780) | | 0.960 (0.159) | | 0.950 (0.663) |
| **Number of non-hydrogen atoms** | 20978 | | 2595 | | 2592 |
| macromolecules | 20005 | | 2537 | | 2368 |
| ligands | 232 | | 29 | | 29 |
| solvent | 741 | | 29 | | 195 |
| Protein residues | 2700 | | 326 | | 325 |
| RMS(bonds) | 0.007 | | 0.012 | | 0.014 |
| RMS(angles) | 0.89 | | 1.63 | | 1.68 |
| Ramachandran favored (%) | 96.29 | | 97.83 | | 97.20 |
| Ramachandran allowed (%) | 3.63 | | 2.17 | | 2.49 |
| Ramachandran outliers (%) | 0.08 | | 0.00 | | 0.31 |
| Rotamer outliers (%) | 3.96 | | 5.73 | | 5.02 |
| Clashscore | 12.60 | | 1.75 | | 2.08 |
| Average B-factor | 27.56 | | 76.39 | | 40.60 |
| macromolecules | 27.60 | | 76.57 | | 40.37 |
| ligands | 22.22 | | 74.72 | | 32.17 |
| solvent | 28.30 | | 62.26 | | 44.64 |

Statistics for the highest-resolution shell are shown in parentheses.

**Supplementary Table S3:** Analysis of key selectivity residues in GuaB across the phylogeny of microbial life. Species were chosen for analysis based on selecting representative species from each major clade from the phylogeny of Hug et al.^32^ aligned using CLUSTAL_MUSCLE software, and classified using NCBI taxonomy website. The data in this table were used to assign A/Y and S/L pairing of key selectivity residues mapped onto the phylogeny of Hug et al.^32^ depicted in Figure 2a. Refer to the separate excel spreadsheet for Supplementary Table S3.

**Supplementary Table S4.** Guanine rescue of GuaB inhibitor activity against *Acinetobacter baumannii* ATCC19606. Minimal inhibitory concentration (MIC) assays were performed according to the Clinical & Laboratory Standards Institute (CLSI) microtiter plate protocol with modification of the media. GuaBi assays using M9 media, or M9 media plus 100 μM guanine were prepared containing serial dilutions (0.1-100μM) of GuaB-inhibitors G1, G2, or G8. Assay plates were inoculated with 5E5 CFU/ml bacteria by colony suspension, and the growth of bacteria was read visually after incubation for 24 hours at 37C. Data are the average of 3 independent experiments.

| ***A.b.*19606 Average CLSI MIC (μM) (n=3)** | | | | | |
| --- | --- | --- | --- | --- | --- |
| **M9 media** | | | **M9 media + 100μM guanine** | | |
| **G2** | **G1** | **G8** | **G2** | **G1** | **G8** |
| 4.2 | 1.0 | 0.4 | >100 | >100 | >100 |

**Supplementary Table S5.** GuaB *in vitro* safety assays.

| **GuaB In Vitro Safety Assays - Compound ID** | **G2** | | **G1** | **G8** |
| --- | --- | --- | --- | --- |
| **Structure** | 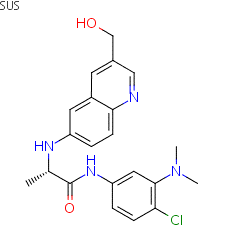 | | 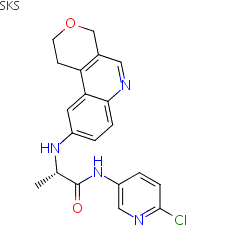 | 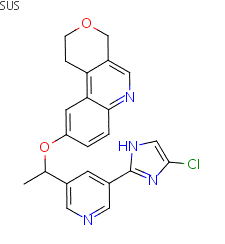 |
| **Secondary Pharmacology at indicated concentration** | 10µM | 50µM | 10µM | 50µM |
| Total Targets | 41 | 41 | 41 | 41 |
| Hits (>50% Binding) | 0 | 1 | 0 | 21 |
| Hits (>75% Binding) | 0 | 0 | 0 | 7 |
| Selectivity Risk | 0% | 3% | 0% | 17% |
| Promiscuity Risk | 0% | 0% | 0% | 51% |
| **hERG 2-point Automatic Patch Clamp Assay** |  |  |  |  |
| hERG 1µM | 1.70% | | 2.80% | - |
| hERG 10µM | 8.60% | | 37.20% | 6.3% |
| hERG 50µM | *48.5% | | - | 50.1% |
| **General Cytotoxicity Assessment 2-day Primary Human Hepatocyte Assay** |  |  |  |  |
| Serum (µM) | IC50>100 | | IC50>100 | IC50 = 19.2 |
| Serum-Free (µM) | IC50>100 | | IC50>100 | IC50 = 30.2 |
| *=Triggered solubility assay - indicating may be a solubility issue at that concentration |  |  |  |  |

**Supplementary Table S6.** Frequency of spontaneous resistance studies in *A. baumannii* 19606 for GuaB inhibitors G1 and G2. Fluctuation analyses performed on 20 independent cultures of bacteria were plated on M9 agar plates with 4x or 8x MIC of the GuaBi, grown for 3 days at 37°C, and number of resistant colonies per plate enumerated. The frequency of resistance was calculated using the P0 method of Foster^44^. Resistant colonies were re-streaked on antibiotic containing plates and sequenced by colony PCR for the *guaB* open reading frame and 500bp flanking region to identify missense mutations in the *guaB* gene. Resistant mutants were characterized for minimal inhibitory concentration (MIC) using the Clinical & Laboratory Standards Institute (CLSI) microtiter protocol, and reported also as the fold-shift in MIC of the mutant bacteria compared to the parental wild-type control.

| **Date of study** | **GuaBi** | **Selection condition** | **F.O.R.** | **Resistance alleles** | **MIC (µM)** | **Fold shift** |
| --- | --- | --- | --- | --- | --- | --- |
| 20180828 | G1 | 4xMIC (3.1µM) | 2.90E-10 | n.d. | n.d. | n.d. |
| 20180821 | G1 | 4xMIC (3.1µM) | 6.38E-11 | Y445S, Y445N | 50, 50 | 64, 64 |
| 20180815 | G1 | 4xMIC (3.1µM) | 9.50E-11 | Y445N | 50 | 64 |
| 20180124 | G2 | 4xMIC (6.4µM) | 3.60E-10 | Y445C, P25A, P25S | 50, 50, 50 | 32, 32, 32 |
| 20180124 | G2 | 8xMIC (12.8µM) | 5.10E-10 | Y445S, Y445C, P25S | 100, 100, 50 | 64, 64, 32 |
| Frequency of resistance (F.O.R.) was determined using the P0 method. | | | | |  |  |
| n.d. = not determined | |  |  |  |  |  |

**Supplementary Table S7.** Cross-resistance of *Acinetobacter baumannii* ATCC19606 GuaB mutants to different GuaB inhibitors. Minimal inhibitory concentration (MIC) assays were performed according to the Clinical & Laboratory Standards Institute (CLSI) microtiter plate protocol with modification. GuaBi MIC assays using M9 media were prepared containing serial dilutions (0.1-100μM) of GuaB-inhibitors G1, G2, or G8. Assay plates were inoculated with 5E5 CFU/ml *Acinetobacter baumannii* ATCC19606 wild-type, or mutant (*guaB:* Y445N, Y445C, Y445S, P25A, or P25S) bacteria by colony suspension, and the growth of bacteria was read visually after incubation for 24 hours at 37C. Data are the average of 3 independent experiments.

|  |  |  |  |
| --- | --- | --- | --- |
|  | ***A.b.*19606 Average CLSI MIC (μM) (n=3)** | | |
| ***A.b.*19606 strain *guaB* allele** | **G2** | **G1** | **G8** |
| **wild-type** | 4.2 | 0.5 | 0.3 |
| **Y445N** | >100 | 41.7 | 1.8 |
| **Y445C** | 50.0 | 1.6 | 0.2 |
| **Y445S** | >100 | >100 | 8.3 |
| **P25A** | 66.7 | 6.3 | 0.8 |
| **P25S** | 66.7 | 2.6 | 0.6 |
|  |  |  |  |
|  | **GuaBi CLSI MIC fold change from parent strain** | | |
| ***A.b.*19606 strain *guaB* allele** | **G2** | **G1** | **G8** |
| **wild-type** | 1 | 1 | 1 |
| **Y445N** | >25 | 80 | 7 |
| **Y445C** | 12 | 3 | 1 |
| **Y445S** | >25 | >200 | 32 |
| **P25A** | 16 | 12 | 3.3 |
| **P25S** | 16 | 5 | 2.5 |
